# Pan-Cancer Analysis Reveals the Multidimensional Expression and Prognostic and Immunologic Roles of *VSTM2L* in Cancer

**DOI:** 10.3389/fmolb.2021.792154

**Published:** 2022-01-27

**Authors:** Shuyi Zhang, Hailin Xiong, Jiahui Yang, Xia Yuan

**Affiliations:** ^1^ Department of Oncology, Huizhou Municipal Central Hospital, Huizhou, China; ^2^ Prenatal Diagnosis Center, Huizhou Municipal Central Hospital, Huizhou, China

**Keywords:** *VSTM2L*, prognosis, immune cell infiltration, immunomodulators, pan-cancer

## Abstract

Immunotherapy can improve survival in a variety of cancers by modulating the interaction between tumors and the tumor immune microenvironment (TIME). V-set and transmembrane domain containing 2 like (*VSTM2L*) regulates interleukin (IL)-4 signaling pathway—which involves immune-related factors—and has been linked to some cancers. However, the expression profile and prognostic significance of *VSTM2L* in different cancers as well as its relationship to the TIME are not known. This study investigated the pan-cancer expression profile, prognostic value, and immunologic relevance of *VSTM2L*. *VSTM2L* expression in different cancers was analyzed using the Cancer Cell Line Encyclopedia (CCLE), Human Protein Atlas (HPA), Tumor Immune Estimation Resource (TIMER), The Cancer Genome Atlas (TCGA), and Genotype–Tissue Expression (GTEx) portal. We examined the association between VSTM2L expression and clinical outcomes by Kaplan–Meier and Cox regression analyses using TCGA and Kaplan–Meier Plotter, and the results were validated in a Gene Expression Omnibus cohort. The correlations between *VSTM2L* expression and immune cell infiltration, immunomodulators, tumor mutation burden (TMB), microsatellite instability (MSI), and immune and stromal scores across cancers were analyzed using TCGA, TIMER, and Tumor–Immune System Interactions and Drugbank databases (TISIDB). The results showed that *VSTM2L* expression varied across cancers and its aberrant expression was associated with clinical outcomes: upregulation of *VSTM2L* was positively associated with advanced stage and reduced overall survival (OS), disease-specific survival (DSS), progression-free interval (PFI), and disease-free interval (DFI) in stomach adenocarcinoma (STAD); and its upregulation was associated with early-stage disease and improved OS, DSS, PFI, and DFI in kidney renal papillary cell carcinoma (KIRP). *VSTM2L* expression level was correlated with immune cell infiltration, expression of immunomodulators, TMB, MSI, and immune and stromal scores in multiple cancers. In conclusion, *VSTM2L* has prognostic value in various cancers and can predict both poor (STAD) and good (KIRP) outcomes. The relationship between *VSTM2L* expression and immune markers suggests a role in modulating the TIME.

## Introduction

The interaction between tumors and the tumor immune microenvironment (TIME) influences the occurrence, progression, and treatment of cancers (Hinshaw and Shevde, 2019; Lei et al., 2020). Although cancer immunotherapy has improved the survival of cancer patients, treatment response rates are low (Binnewies et al., 2018). Clarifying tumor–TIME interactions can help to identify novel markers for predicting the response to immunotherapy or that can serve as pharmacologic targets (Binnewies et al., 2018; Taube et al., 2018; Petitprez et al., 2020).

V-set and transmembrane domain-containing two like (*VSTM2L*, also known as C20orf102) is expressed in multiple human tissues, with the highest expression observed in the cerebral cortex and pituitary. As a secreted protein that antagonizes the neuroprotective peptide humanin, *VSTM2L* has been implicated in neurodegenerative and metabolic diseases (Rossini et al., 2011). However, there have been few studies on the role of *VSTM2L* in cancer. *VSTM2L* was shown to be downregulated in Helicobacter pylori-positive gastric cancer compared to corresponding normal tissues (Hu et al., 2018); additionally, the CpG island methylation phenotype (CIMP)-related gene signature comprising *VSTM2L* and five other genes showed prognostic value in gastric cancer (Zeng et al., 2020). In locally advanced rectal cancer, elevated expression of *VSTM2L* conferred chemoradiotherapy resistance via regulation of interleukin (IL)-4 signaling pathway (Liu et al., 2021), which is involved in the immune response in cancer patients (Lee et al., 2009; Rajaraman et al., 2009; Siliņa et al., 2011; Li et al., 2019; Didonna et al., 2020; Wei et al., 2020).

The above observations suggest that *VSTM2L* plays an important role in cancer and likely modulates immunity, although this has not yet been reported. To address this point, in this study we used multiple databases, including TCGA, GEO, CCLE, HPA, TIMER, GTEx, and TISIDB in combination with Kaplan–Meier Plotter to perform a comprehensive and multidimensional pan-cancer analysis of the role of *VSTM2L*. We compared *VSTM2L* expression in different types of cancer and corresponding normal tissue. We also evaluated the prognostic value of *VSTM2L* in cancer and investigated the relationship between *VSTM2L* expression level and various aspects of the TIME including immune cell infiltration, expression of immunomodulators (such as immunostimulators, immunoinhibitors, and major histocompatibility complex (MHC) molecules), tumor mutation burden (TMB), microsatellite instability (MSI), and Immune/StromalScores.

## Materials and Methods

### Data Collection and Processing

*VSTM2L* expression level in human cancer cell lines was determined from RNA sequencing (RNA-seq) data in the Cancer Cell Line Encyclopedia (CCLE) database (https://portals.broadinstitute.org/ccle). The expression module in Tumor Immune Estimation Resource (TIMER; https://cistrome.shinyapps.io/timer/) was used to visualize differential expression of *VSTM2L* across 33 tumors in The Cancer Genome Atlas (TCGA) compared to adjacent normal tissue, including adrenocortical carcinoma (ACC), bladder urothelial carcinoma (BLCA), breast invasive carcinoma (BRCA), cervical squamous cell carcinoma (CESC), cholangiocarcinoma (CHOL), colon adenocarcinoma (COAD), lymphoid neoplasm diffuse large B cell lymphoma (DLBC), esophageal carcinoma (ESCA), glioblastoma multiforme (GBM), brain lower grade glioma (LGG), head and neck squamous cell carcinoma (HNSC), kidney chromophobe (KICH), kidney renal clear cell carcinoma (KIRC), kidney renal papillary cell carcinoma (KIRP), acute myeloid leukemia, liver hepatocellular carcinoma (LIHC), lung adenocarcinoma (LUAD), lung squamous cell carcinoma (LUSC), mesothelioma (MESO), ovarian serous cystadenocarcinoma (OV), pancreatic adenocarcinoma (PAAD), pheochromocytoma and paraganglioma (PCPG), prostate adenocarcinoma (PRAD), rectum adenocarcinoma (READ), sarcoma (SARC), skin cutaneous melanoma (SKCM), stomach adenocarcinoma (STAD), testicular germ cell tumors (TGCT), thyroid carcinoma (THCA), thymoma (THYM), uterine corpus endometrial carcinoma (UCEC), uterine carcinosarcoma (UCS), and uveal melanoma (UVM). Additionally, *VSTM2L* expression data of 31 normal tissues were obtained from the Genotype–Tissue Expression (GTEx) database (https://commonfund.nih.gov/GTex), and data for 33 cancer types, adjacent normal tissue, and corresponding clinical information of patients were extracted from TCGA datasets obtained from the University of California Santa Cruz Xena browser (https://xena.ucsc.edu/). Differential expression analyses of *VSTM2L* between cancer and normal tissues were performed by merging the mRNA expression data from GTEx with TCGA datasets, and data on protein expression detected by immunohistochemistry was obtained from Human Protein Atlas (HPA). *VSTM2L* expression was also evaluated with respect to clinical stage in 33 cancers. Whole RNA-seq data were normalized through log2 conversion.

### Evaluation of Prognostic Utility

Kaplan–Meier survival and Cox regression analyses were used to evaluate the prognostic value of *VSTM2L* expression for overall survival (OS), disease-specific survival (DSS), progression-free interval (PFI), and disease-free interval (DFI) in 33 cancer types using the “survival” and “survminer” packages of R software. According to the median *VSTM2L* expression in each cancer type, patients were divided into high and low expression groups and Kaplan–Meier survival analysis was carried out using prognostic data. Kaplan–Meier Plotter (http://kmplot.com/analysis/) was used to examine the influence of *VSTM2L* on OS in patients with STAD or KIRP based on clinicopathologic factors and cell content, respectively. Gene Expression Omnibus (GEO) data (GSE84437 and GSE2748) were used to assess the prognostic value of *VSTM2L* in STAD and KIRP. Hazard ratio (HR) with 95% confidence intervals (CIs) and the log-rank *p* value were determined by Cox regression analysis. A log *p* value < .05 was considered statistically significant.

### Immune Correlation Analysis

We used TIMER (https://cistrome.shinyapps.io/timer/) to quantify the association between *VSTM2L* expression in diverse cancer types and the abundance of six tumor-infiltrating immune cells (TIIC) types including B cells, cluster of differentiation (CD)8 + T cells, CD4^+^ T cells, macrophages, neutrophils, and dendritic cells in the tumor mass as well as other immune cells, which was adjusted by purity. Gene lists of three types of immunomodulator including immunoinhibitors, immunostimulators, and MHC molecules were obtained from the Tumor–immune System Interactions and Drugbank (TISIDB) database (http://cis.hku.hk/TISIDB/index.php). TIMER was used to examine the correlation between *VSTM2L* expression level and immunomodulator genes in various cancers. Spearman correlations between *VSTM2L* expression and immune and stromal scores (ImmuneScore and StromalScore, respectively) were determined.

### TMB and MSI Correlation Analyses

TMB was defined as the total incidences of mutation per million base pairs and MSI was calculated as the number of deletion or insertion events occurring in repeating sequences of genes; both were obtained from TCGA. The Spearman correlation test was used to determine the correlations between *VSTM2L* expression and TMB and MSI in 33 cancers types.

### Statistical Analysis

*VSTM2L* expression levels in tumor and normal tissues across cancers were compared by wilcoxon test. Kaplan-Meier analysis and univariate Cox regression analysis were used to evaluate the correlation between *VSTM2L* expression and patient prognosis. Correlations between *VSTM2L* expression and TIICs, immunomodulators, ImmuneScore, StromalScore, TMB, and MSI were determined by Spearman correlation analysis. The threshold for statistical significance was set as *p* < .05.

## Results

### Aberrant Expression of *VSTM2L* in Cancers

**FIGURE 1:**
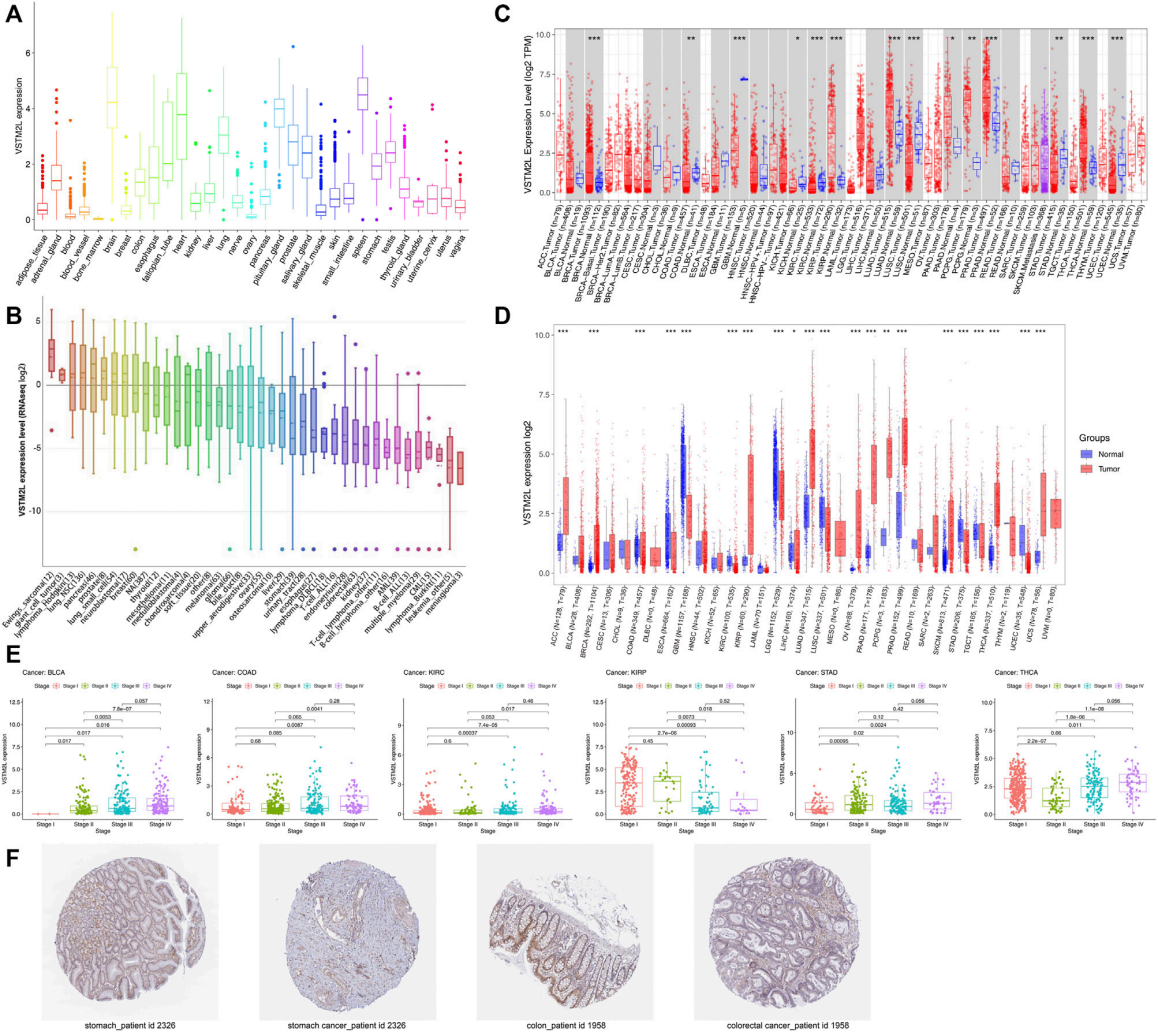
*VSTM2L* expression in different cancers. **(A)** RNA-seq data of *VSTM2L* expression level in human cancer cell lines in the CCLE database. **(B)** *VSTM2L* expression data of 31 normal tissues from GTEx datasets. **(C)** Differential expression of *VSTM2L* in various cancer types based on TCGA data in TIMER. **(D)** *VSTM2L* expression level in 33 cancer types from combined GTE and TCGA data. **(E)** Relationship between *VSTM2L* expression level and clinical stage in BLCA, COAD, KIRC, KIRP, STAD, and THCA. **(F)** Immunohistochemical detection of *VSTM2L* protein expression in the tumor and corresponding normal tissue of a STAD patient (ID: 2,326) and COAD patient (ID: 1958) in the HPA database. *p < .05, **p < .01, ***p < .001.

GTEx data showed that *VSTM2L* mRNA was widely expressed in normal tissues, with the highest expression in spleen, brain, and pituitary and the lowest expression in bone marrow and blood (Figure 1A). RNA-seq data in the CCLE database showed that *VSTM2L* was highly expressed in Ewing sarcoma cell line (Figure 1B). We analyzed *VSTM2L* expression in different cancers; the TIMER data across all TCGA tumors showed that compared to corresponding normal tissue, *VSTM2L* was significantly upregulated in BRCA, KIRP, LUAD, PAAD, PCPG, PRAD, and THCA and downregulated in COAD, GBM, KICH, KIRC, LUSC, STAD, and UCEC (Figure 1C). Analysis of combined TCGA and GTEx data revealed similar trends in *VSTM2L* expression as observed in the TIMER data, but it also showed that *VSTM2L* was significantly upregulated in ACC, OV, SKCM, and UCS and downregulated in ESCA, LGG, LIHC, and TGCT relative to matched normal tissue (Figure 1D). Advanced tumor stage was more closely associated with *VSTM2L* expression in BLCA, COAD, KIRC, STAD, and THCA, while the opposite was true for KIRP (Figure 1E). No association was found between *VSTM2L* expression and cancer stage in other cancer types (Supplementary Figure S1). *VSTM2L* protein expression in STAD and COAD and corresponding normal tissue detected by immunohistochemistry was determined from HPA datasets. Consistent with the mRNA levels, *VSTM2L* showed lower expression in both gastric cancer and colon cancer tissues than in normal tissues (Figure 1F).

### Prognostic Value of *VSTM2L* in Cancers

**FIGURE 2:**
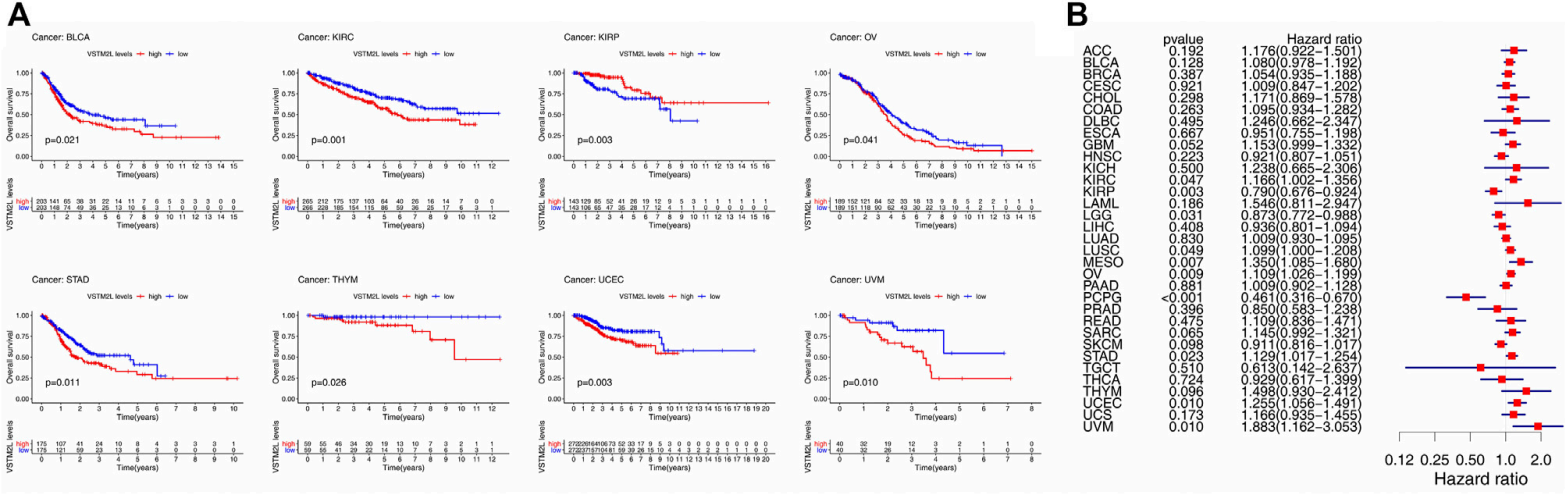
Kaplan–Meier survival and Cox regression analyses of the prognostic value of *VSTM2L* expression level for OS in different cancer types. **(A)** OS according to high and low *VSTM2L* expression in BLCA, KIRC, KIRP, OV, STAD, THYM, UCEC, and UVM from TCGA database. **(B)** Correlation between *VSTM2L* mRNA expression level and OS in various cancer types. *p < 0.05.

The Kaplan–Meier survival and Cox regression analyses based on TCGA data showed that the prognostic value of *VSTM2L* differed according to cancer types. High *VSTM2L* expression was associated with shorter OS in BLCA, KIRC, OV, STAD, THYM, UCEC, and UVM but predicted better outcome in KIRP (Figure 2A); *VSTM2L* expression had no prognostic value in the OS of other cancers (Supplementary Figure S2). Cox regression analysis showed that increased *VSTM2L* expression was correlated with shorter OS in KIRC (*p* = .047, HR = 1.166), LUSC (*p* = .049, HR = 1.099), MESO (*p* = .007, HR = 1.350), OV (*p* = .009 HR = 1.109), STAD (*p* = .023, HR = 1.129), UCEC (*p* = .010, HR = 1.255), and UVM (*p* = .010, HR = 1.883) (Figure 2B) and predicted a better outcome in KIRP (*p* = .003, HR = .790), LGG (*p* = .031, HR = .873), and PCPG (*p* < .001, HR = .461) (Figure 2B).

**FIGURE 3:**
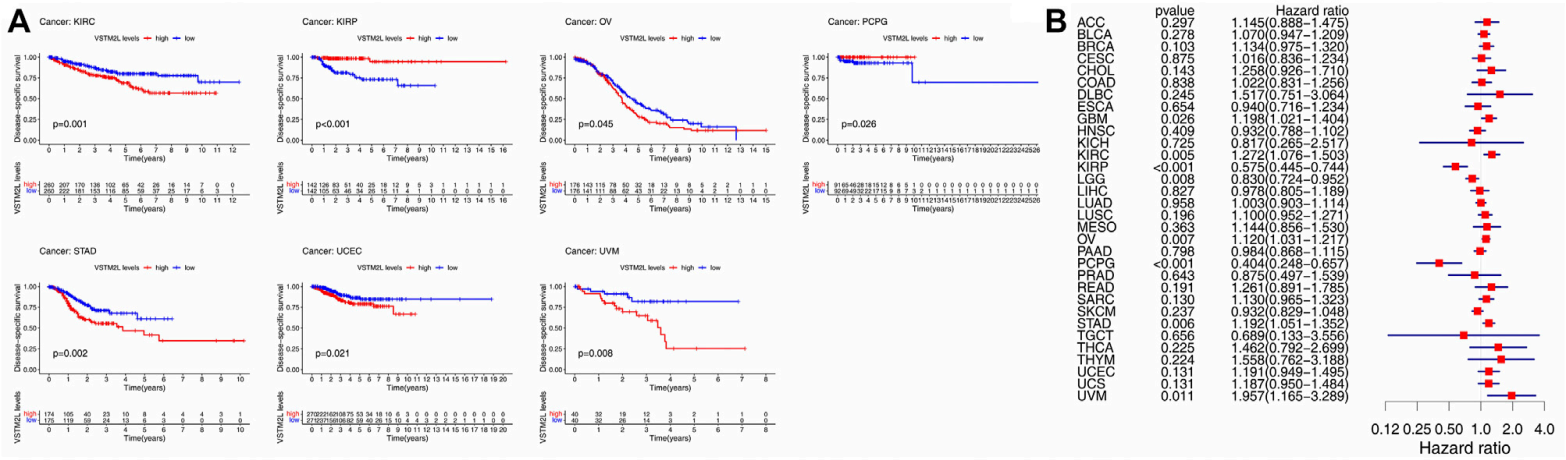
Kaplan–Meier survival and Cox regression analyses of the prognostic value of *VSTM2L* expression level for DSS in different cancer types. **(A)** DSS according to high and low *VSTM2L* expression in KIRC, KIRP, OV, PCPG, STAD, UCEC, and UVM from TCGA. **(B)** Correlation between *VSTM2L* mRNA expression level and DSS in various cancer types. *p < .05.

High *VSTM2L* expression was associated with shorter DSS in KIRC, OV, STAD, UCEC, and UVM and longer DSS in KIRP and PCPG in the Kaplan–Meier survival analysis (Figure 3A). The same results were obtained by Cox regression analysis, which also revealed a significant HR for GBM and LGG (Figure 3B).

**FIGURE 4:**
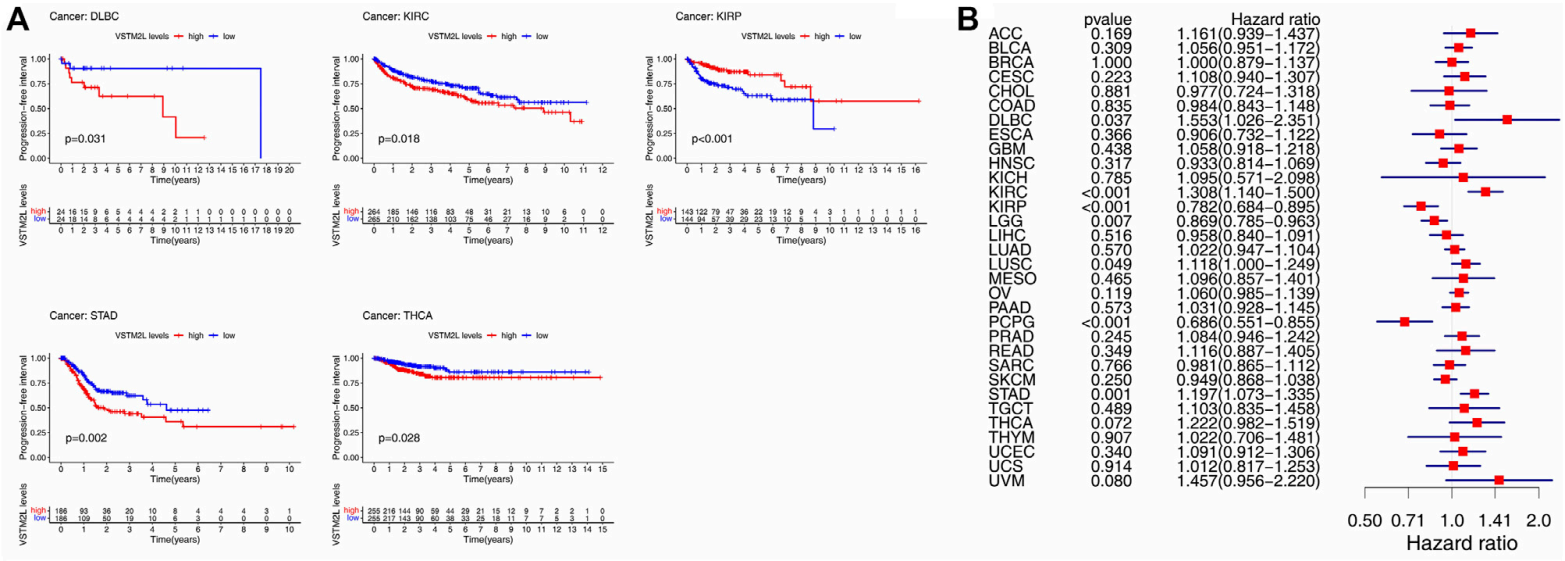
Kaplan–Meier survival and Cox regression analyses of the prognostic value of *VSTM2L* expression level for PFI in different cancer types. **(A)** PFI according to high and low *VSTM2L* expression in DLBC, KIRC, KIRP, STAD, and THCA from TCGA. **(B)** Correlation between *VSTM2L* mRNA expression level and PFI in various cancer types. *p < .05.

**FIGURE 5:**
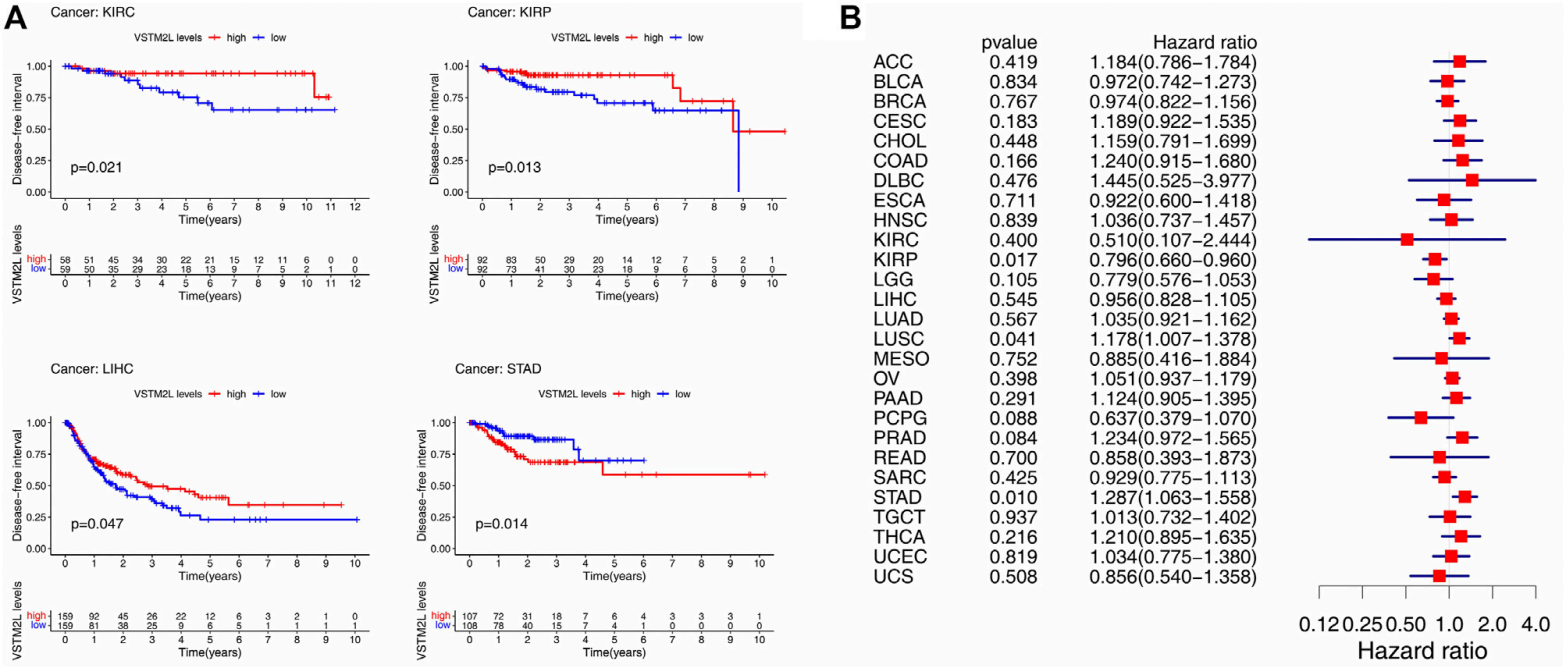
Kaplan–Meier survival and Cox regression analyses of the prognostic value of *VSTM2L* expression level for DFI in different cancer types. **(A)** DFI according to high and low *VSTM2L* expression in KIRC, KIRP, LIHC, and STAD from TCGA. **(B)** Correlation between *VSTM2L* mRNA expression level and DFI in various cancer types. *p < .05.

Elevated *VSTM2L* expression was linked to shorter PFI in DLBC, KIRC, STAD, and THCA, and shorter DFI in STAD; it was also associated with longer PFI in KIRP and longer DFI in KIRC, KIRP, and LIHC (Figure 4A and Figure 5A). Upregulation of *VSTM2L* was correlated with high HRs of PFI in DLBC, KIRC, LUSC, and STAD and high HRs of DFI in LUSC and STAD (Figure 4B and Figure 5B). Meanwhile, downregulation of *VSTM2L* expression was correlated with high HRs of PFI in KIRP, LGG, and PCPG and high HRs of DFI in KIRP.

### Relationship Between *VSTM2L* Expression and Immune Cell Infiltration and Immunomodulator Expression in Different Cancers

**FIGURE 6:**
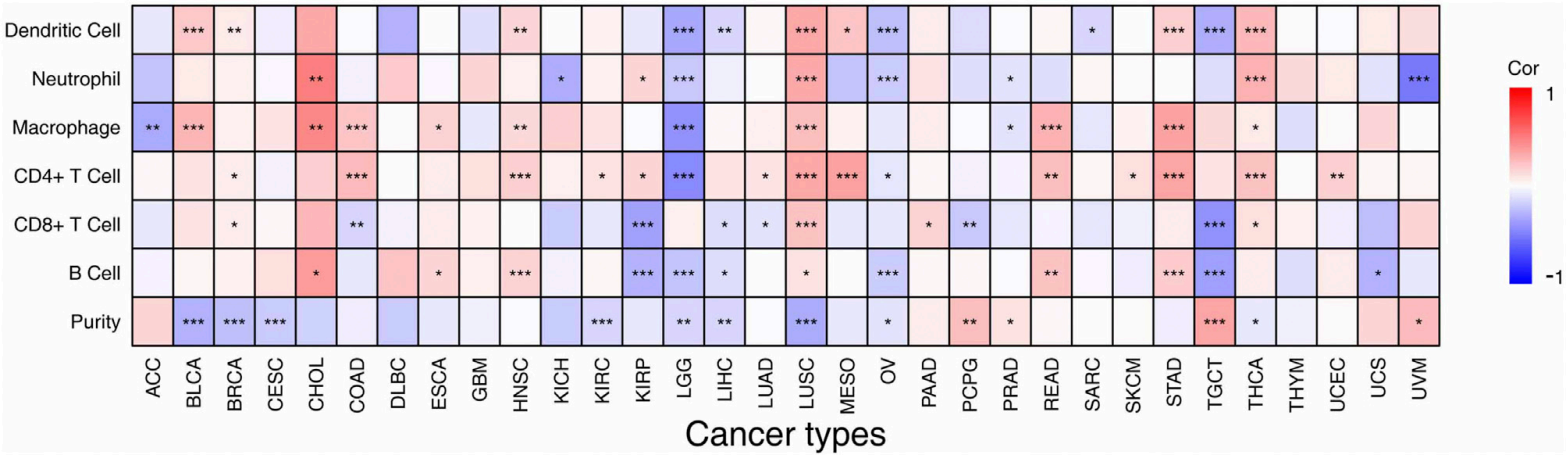
Association between *VSTM2L* expression level and abundance of TIICs in different cancer types in TIMER, shown as a purity-corrected partial Spearman’s rho value. *P_*adjusted*_ < .05, **P_*adjusted*_ < .01, ***P_*adjusted*_ < .001.

As the TIME is linked to prognosis and response to immunotherapy in cancer (Binnewies et al., 2018; Hinshaw and Shevde, 2019), we next examined the relationship between *VSTM2L* expression and immune cell infiltration (Figure 6, details are shown in Supplementary Figure S3) and the expression of immunomodulators in the TIME across 33 cancers extracted from TCGA datasets using TIMER. We found that *VSTM2L* was related to TIICs in most cancers with the exception of DLBC, GBM, and THYM. In CHOL and LUSC, *VSTM2L* expression was positively correlated with immune cell infiltration. Specifically, *VSTM2L* was positively correlated with B cells (r = .38, *p* = .0239), macrophages (r = .46, *p* = .0059), and neutrophils (r = .48, *p* = .0033) in CHOL and with CD4^+^ T cells (r = .33, *p* = 7.78E-14), neutrophils (r = .32, *p* = 4.54E-13), and dendritic cells (r = .34, *p* = 5.57E-14) in LUSC. On the contrary, *VSTM2L* was negatively correlated with CD4^+^ T cells (r = −.44, *p* = 3.88E-24), macrophages (r = −.42, *p* = 2.26E-21), and dendritic cells (r = −.33, *p* = 7.49E-14) in LGG and with B cells (r = −.36, *p* = 7.50E-06), CD8^+^ T cells (r = −.41, *p* = 1.97E-07), and dendritic cells (r = −.30, *p* = .0002) in TGCT.

**FIGURE 7:**
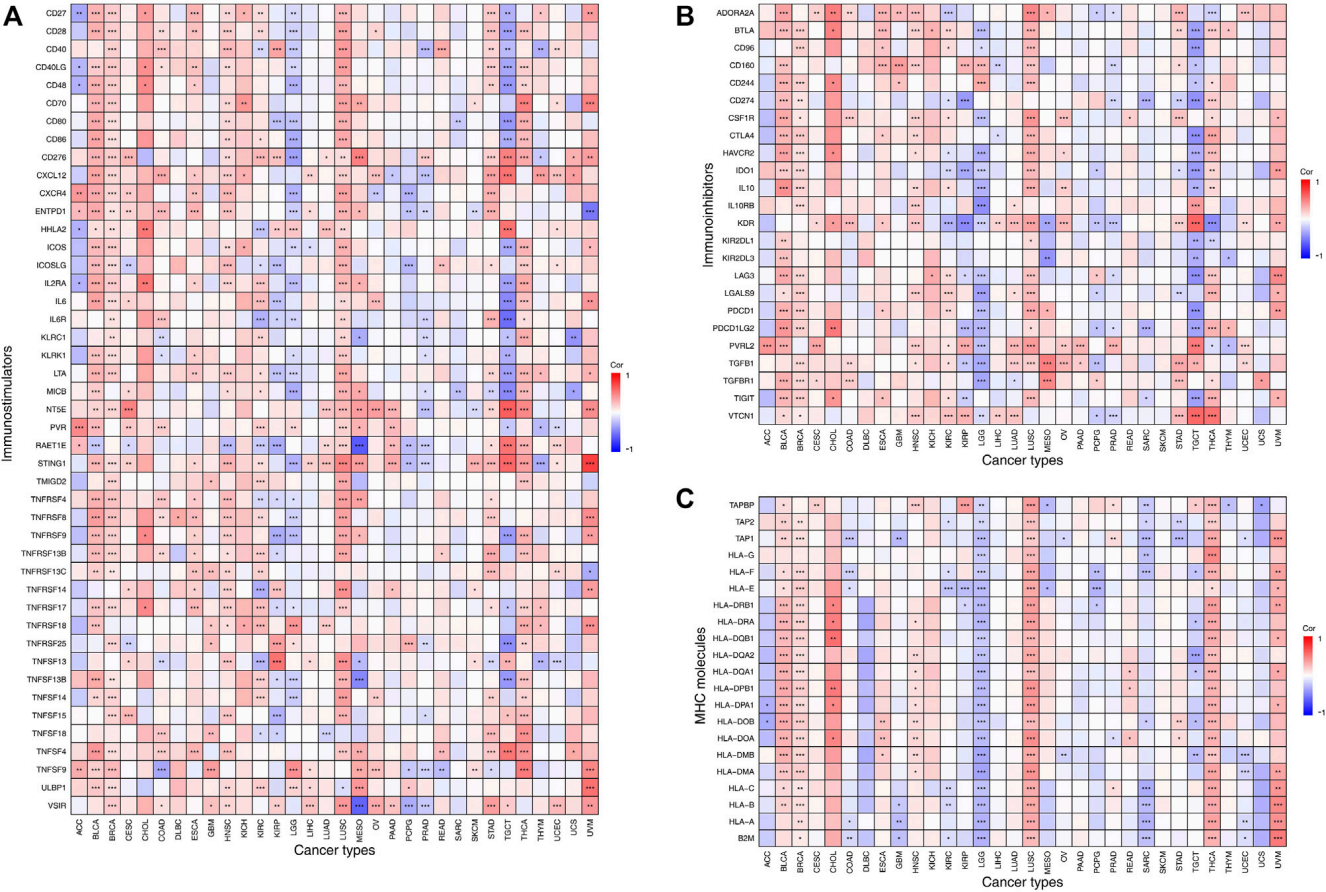
Association between expression levels of *VSTM2L* and immune checkpoint markers in TIMER, which includes immune checkpoint genes from the TISIDB database. Association between the expression of *VSTM2L* and immunostimulatory factors **(A)** and immunoinhibitory factors **(B)** and MHC molecules **(C)**. *P_*adjusted*_ < .05, **P_*adjusted*_ < .01, ***P_*adjusted*_ < .001.

We next examined the correlation between the expression of *VSTM2L* and immunomodulator genes including immunostimulators (Figure 7A, details are shown in Supplementary Figure S4), immunoinhibitors (Figure 7B, details are shown in Supplementary Figure S5), and MHC molecules (Figure 7C, details are shown in Supplementary Figure S6) in 33 tumors from the TISIDB database. *VSTM2L* expression was positively associated with that of immune checkpoint markers as well as immunostimulators in BLCA, BRCA, ESCA, HNSC, LUSC, STAD, and THCA, whereas a negative association was observed in KIRP and LGG. In particular, there was a complex relationship between *VSTM2L* and immunostimulators in TGCT (STING1: r = .59, *p* = 3.70E-14; NT5E: r = .62, *p* = 1.72E-16; IL6R: r = −.56, *p* = 5.58E-12), MESO (RAET1E: r = −.63, *p* = 7.74E-10; VSIR: r = −.60, *p* = 9.20E-09), and UVM (STING1: r = .73, *p* = 1.40E-13; ULBP1: r = .55, *p* = 6.49E-06); and between *VSTM2L* and immunoinhibitors in CHOL (PDCD1LG2: r = 0.52, *p* = .0054, ADORA2A: r = .49, *p* = .0071), TGCT (VTCN1: r = .58, *p* = 8.33E-14, PVRL2: r = .51, *p* = 4.03E-10, KDR: r = .60, *p* = 9.99E-15), THCA (VTCN1: r = .54, *p* = 9.62E-39), and MESO (TGFB1: r = .50, *p* = 6.50E-06). Immunoinhibitors that are targets of immunotherapies and were found here to be associated with *VSTM2L* in various cancers included CD274, also known as programmed death-ligand 1(PD-L1) (KIRP: r = −.38, *p* = 6.39E-10; TGCT: r = −.42, *p* = 2.05E-06); cytotoxic T lymphocyte-associated protein 4(CTLA-4) (TGCT: r = −.43, *p* = 2.97E-07); and PDCD1, also known as programmed death (PD)-1 (TGCT: r = −.42, *p* = 6.15E-07; UVM: r = .38, *p* = .0029). We also observed that *VSTM2L* expression was positively correlated with MHC molecules in BLCA, BRCA, CHOL, HNSC, LUSC, THCA, and UVM, while a negative association was observed in KIRC, LGG, SARC, and TGCT.

### Correlation Between *VSTM2L* Expression and ImmuneScore, StromalScore, TMB and MSI in Cancers

**FIGURE 8:**
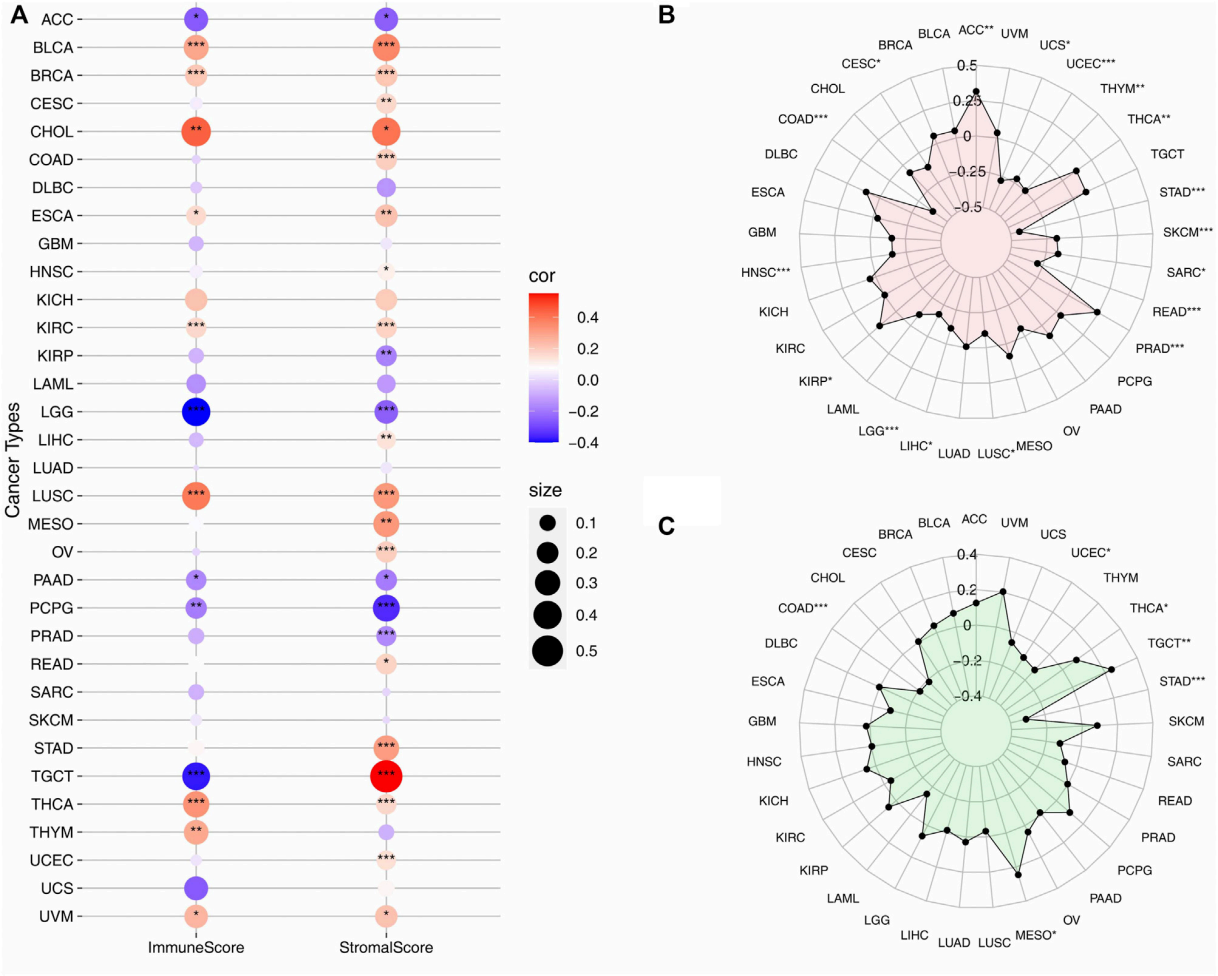
Correlation between *VSTM2L* expression level and markers of the TIME and tumor prognosis. **(A)** Correlation between *VSTM2L* expression and ImmuneScore and StromalScore. The color and area of each circle represent the degree and direction of correlation, with larger circles corresponding to a stronger correlation and red and blue corresponding to a positive and negative correlation, respectively. **(B,C)** Correlation between *VSTM2L* expression level and TMB **(B)** and MSI **(C)**. *p < .05, **p < .01, ***p < .001.

*VSTM2L* expression in BLCA, BRCA, CHOL, ESCA, KIRC, LUSC, THCA, and UVM was positively correlated with ImmuneScore and StromalScore; and was negatively correlated with these scores in ACC, LGG, PAAD, and PCPG. Additionally, in TGCT, *VSTM2L* expression was negatively and positively correlated with ImmuneScore and StromalScore, respectively. The three cancer types showing the strongest correlation between *VSTM2L* expression and ImmuneScore were CHOL (r = .44, *p* = .0084), LGG (r = −.4, *p* = 2.20E-16), and LUSC (r = .39, *p* = 2.20E-16); and the top three cancers related to StromalScore were TGCT (r = .55, *p* = 2.20E-16), CHOL (r = .4, *p* = .0158), and BLCA (r = .36, *p* = 6.44E-14) (Figure 8A, details are shown in Supplementary Figure S7).

TMB and MSI are useful prognostic markers and can predict the response to immunotherapy in various cancer types (Dudley et al., 2016; Chan et al., 2019; Franke et al., 2019). We examined the correlation between *VSTM2L* expression and TMB (Figure 8B, details are shown in Supplementary Figure S8) or MSI (Figure 8C, details are shown in Supplementary Figure S8) across 33 cancers and found that it was positively correlated with both markers in THCA and negatively correlated these markers in COAD, STAD, and UCEC. Significant correlations were also observed between *VSTM2L* upregulation and decreased TMB in other 10 cancer types (CESC, HNSC, LGG, LIHC, LUSC, READ, SARC, SKCM, THYM, and UCS) and increased TMB in four cancer types (ACC, KIRP, PRAD, and THCA). We also found that MESO and TGCT patients with high *VSTM2L* expression were more likely to have high MSI.

### Identification of *VSTM2L* as a Key Marker in STAD and KIRP

In order to evaluate the prognostic value of *VSTM2L* in STAD and KIRP, we examined survival data from the Kaplan–Meier Plotter database in relation to clinicopathologic factors (Table 1) and immune cell profile (Table 2). High *VSTM2L* expression level was correlated with worse OS in STAD patients who were female (HR = 1.86, *p* = .034), male (HR = 1.88, *p* = .0044), stage 3 (HR = 2.22, *p* = .0102), grade 2 (HR = 2.27, *p* = .0051), grade 3 (HR = 1.79, *p* = 0.0176), White (HR = 1.7, *p* = .0121), or Asian (HR = 3.87, *p* = .0029) or those with high TMB (HR = 1.89, *p* = .0083). On the other hand, high *VSTM2L* expression level was correlated with better OS in KIRP patients who were female (HR = .27, *p* = .0129), male (HR = .36, *p* = .0032), stage 3 (HR = .12, *p* = .0158), White (HR = .44, *p* = .0153), or Black/African American (HR = .13, *p* = .0079) or those with low TMB (HR = .21, *p* = .0002), with stage 1 patients (HR = 3.33, *p* = .0297) as an exception.

**TABLE 1:** Correlation of *VSTM2L* expression and overall survival in STAD and KIRP with different clinicopathologic parameters by Kaplan-Meier plotter.

Clinicopathologic parameters	Overall survival of STAD			Overall survival of KIRP		
	N	Hazard ratio	*p*-value	N	Hazard ratio	*p*-value
Sex						
female	133	1.86 (1.04–3.35)	**.034**	76	.27 (.09–0.82)	**.0129**
male	238	1.88 (1.21–2.93)	**.0044**	211	.36 (.18–.73)	**.0032**
Stage						
1	50	2.57 (0.77–8.54)	.1117	171	3.33 (1.06–10.47)	**.0297**
2	111	1.47 (0.74–2.91)	.2681	21	910889238.05 (0 − Inf)	.3599
3	149	2.22 (1.19–4.15)	**.0102**	51	.12 (.02–.93)	**.0158**
4	38	1.83 (0.77–4.33)	.162	15	—	—
Grade						
1	12	—	—	—	—	—
2	134	2.27 (1.26–4.09)	**.0051**	—	—	—
3	218	1.79 (1.1–2.92)	**.0176**	—	—	—
4	0	—	—	—	—	—
Race						
white	237	1.7 (1.12–2.58)	**.0121**	205	.44 (.22–0.87)	**.0153**
asian	73	3.87 (1.49–10.08)	**.0029**	6	—	—
black/african american	13	—	—	60	.13 (.02–.74)	**.0079**
Mutation burden						
high	186	1.89 (1.17–3.06)	**.0083**	141	.53 (.21–1.34)	.1747
low	182	1.64 (0.97–2.78)	.0639	136	.21 (.09–.52)	**.0002**

The *p*-values in bold are statistically significant (less than 0.05).

**TABLE 2:** Correlation of *VSTM2L* expression and overall survival in STAD and KIRP according to the immune cell profile using Kaplan-Meier plotter.

Cellular content	Overall survival of STAD			Overall survival of KIRP		
	N	Hazard ratio	*p*-value	N	Hazard ratio	*p*-value
Basophils						
enriched	66	5.16 (1.52–17.49)	**.0033**	148	.18 (.06–.52)	**.0003**
decreased	303	1.51 (1.05–2.19)	**.027**	137	.37 (.17–.81)	**.0095**
B-cells						
enriched	204	1.79 (1.1–2.9)	**.017**	220	.31 (.14–.65)	**.0012**
decreased	165	2.13 (1.28–3.57)	**.0031**	65	.22 (.07–.63)	**.0021**
CD4^+^ memory T-cells						
enriched	222	1.86 (1.18–2.92)	**.0069**	64	.11 (.01–.82)	**.0088**
decreased	147	1.66 (0.93–2.94)	.081	221	.42 (.2–.85)	**.0132**
CD8^+^ T-cells						
enriched	186	1.33 (0.83–2.14)	.23	146	.53 (.21–1.34)	.1719
decreased	183	2.74 (1.64–4.57)	**5.9e−05**	139	.19 (.08–.42)	**6.7e−06**
Eosinophils						
enriched	277	1.69 (1.13–2.52)	**.0101**	213	.58 (.27–1.25)	.1566
decreased	92	2.55 (1.33–4.87)	**.0033**	72	.09 (.01–.68)	**.0034**
Macrophages						
enriched	193	1.73 (1.07–2.79)	**.0235**	237	.28 (.14–.57)	**.00015**
decreased	176	2.11 (1.26–3.56)	**.0039**	48	.46 (.14–1.54)	.1981
Mesenchymal stem cells						
enriched	150	1.73 (1.05–2.85)	**.03**	121	.25 (.08–.78)	**.01**
decreased	219	2.12 (1.28–3.51)	**.003**	164	.28 (.13–.6)	**.0005**
Natural killer T-cells						
enriched	237	1.97 (1.2–3.25)	**.0066**	149	.26 (.12–.57)	**.0003**
decreased	132	2.38 (1.25–4.52)	**.0064**	136	.43 (.16–1.1)	.0703
Regulatory T-cells						
enriched	272	1.99 (1.33–2.96)	**.0006**	118	.21 (.08–.57)	**.00075**
decreased	97	1.44 (0.71–2.96)	.31	167	.3 (.14–.65)	**.0013**
Type 1 T-helper cells						
enriched	155	2.28 (1.35–3.85)	**.0015**	66	.18 (.06–.56)	**.001**
decreased	214	1.72 (1.01–2.92)	**.0442**	219	.33 (.16–.67)	**.0012**
Type 2 T-helper cells						
enriched	344	1.68 (1.19–2.36)	**.0027**	16	—	—
decreased	25	.36 (.09–1.45)	**.14**	269	.37 (.19–.73)	**.0029**

The *p*-values in bold are statistically significant (less than 0.05).

*VSTM2L* expression level was negatively correlated with the OS of STAD patients with basophil enrichment (HR = 5.16, *p* = .0033) or reduction (HR = 1.51, *p* = .027); B cell enrichment (HR = 1.79, *p* = .017) or reduction (HR = 2.13, *p* = .0031); CD4^+^ memory T cell enrichment (HR = 1.86, *p* = .0069); CD8^+^ T cell reduction (HR = 2.74, *p* = 5.9E-05); eosinophil enrichment (HR = 1.69, *p* = .0101) or reduction (HR = 2.55, *p* = .0033); macrophage enrichment (HR = 1.73, *p* = .0235) or reduction (HR = 2.11, *p* = .0039); mesenchymal stem cell enrichment (HR = 1.73, *p* = .03) or reduction (HR = 2.12, *p* = .003); natural killer T cell enrichment (HR = 1.97, *p* = .0066) or reduction (HR = 2.38, *p* = .0064); regulatory T cell enrichment (HR = 1.99, *p* = .0006); type 1 T helper cell enrichment (HR = 2.28, *p* = .0015) or reduction (HR = 1.72, *p* = .0442); and type 2 T helper cell enrichment (HR = 1.68, *p* = .0027). *VSTM2L* expression was also positively correlated with the OS of KIRP patients with basophil enrichment (HR = .18, *p* = .0003) and reduction (HR = .37, *p* = .0095); B cell enrichment (HR = .31, *p* = .0012) and reduction (HR = .22, *p* = .0021); CD4^+^ memory T cell enrichment (HR = .11, *p* = .0088) and reduction (HR = .42, *p* = .0132); CD8^+^ T cell reduction (HR = .19, *p* = 6.7E-06); eosinophil reduction (HR = .09, *p* = .0034); macrophage enrichment (HR = .28, *p* = .00015); mesenchymal stem cell enrichment (HR = .25, *p* = .01) and reduction (HR = .28, *p* = .0005); natural killer T cell enrichment (HR = .26, *p* = .0003); regulatory T cell enrichment (HR = .21, *p* = .00075) and reduction (HR = .3, *p* = .0013); type 1 T helper cell enrichment (HR = .18, *p* = .001) and reduction (HR = .33, *p* = .0012); and type 2 T helper cell reduction (HR = .37, *p* = .0029).

**FIGURE 9:**
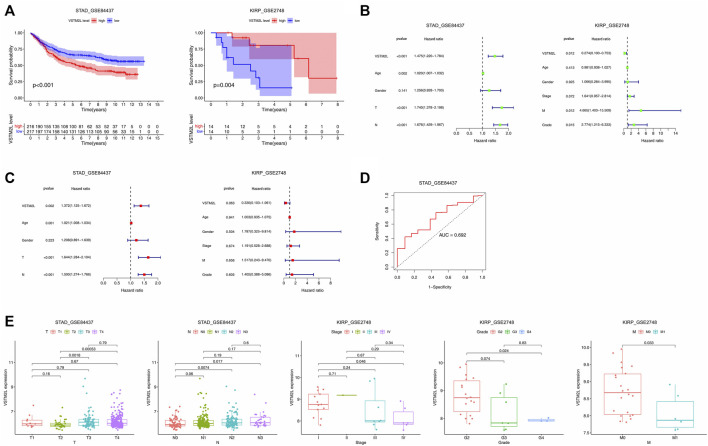
Prognostic value of *VSTM2L* expression in STAD and KIRP. **(A)** OS of STAD patients in the GSE84437 dataset and of KIRP patients in the GSE2748 dataset. **(B,C)** Univariate **(B)** and multivariate **(C)** Cox regression analyses of *VSTM2L* expression level and clinical characteristics of STAD and KIRP patients. **(D)** Time receiver operating characteristic curve (ROC) analysis with area under the ROC curve (AUC) representing the prognostic performance of *VSTM2L* expression for OS in STAD patients. **(E)** Relationship between *VSTM2L* and tumor–node–metastasis stage in STAD and KIRP patients.

GEO cohort data were used to identify potential markers of aberrant *VSTM2L* expression in STAD (GSE84437 dataset) and KIRP (GSE2748 dataset). The Kaplan–Meier survival analysis showed that increased *VSTM2L* expression was associated with poor OS in STAD and with improved OS in KIRP (Figure 9A). Univariate (Figure 9B) and multivariate (Figure 9C) Cox regression analyses showed that *VSTM2L* was an independent prognostic marker for survival when compared to clinical characteristics such as primary tumor and lymph nodes status for patients with STAD, and *VSTM2L* also showed great prognostic potential in KIRP. The time-dependent receiver operating characteristic (ROC) curve analysis of 1-year OS showed that *VSTM2L* had good predictive performance (area under roc curve, AUC = .692) in these patients (Figure 9D). High *VSTM2L* expression was also related to advanced tumor status (T2 vs T3, *p* = .0018; T2 vs. T4, *p* = .00053) and node status (N0 vs N2, *p* = .0074; N0 vs. N3, *p* = .017) in STAD patients irrespective of sex and age. In contrast, in KIRP, high *VSTM2L* expression was related to early tumor stage, better differentiation, and lower rate of metastasis (Figure 9E).

## Discussion

The development of immunotherapy has greatly improved the prognosis of multiple cancers. However, its clinical benefits have not been fully confirmed due to a lack of useful markers (Steven et al., 2016; Sugie, 2018; Ganesh et al., 2019; Hegde and Chen, 2020; Walk et al., 2020). In this study, we found that *VSTM2L* expression differed between tumor and normal tissues and that this was linked to the clinical outcomes in various cancer types. *VSTM2L* was shown to be highly expressed in the cerebral cortex and pituitary and antagonized the neuroprotective peptide humanin (Rossini et al., 2011); this expression pattern was supported by our data. A higher mortality rate was observed in high-risk CIMP patients with elevated expression of prognostic genes including *VSTM2L* (Zeng et al., 2020); and in rectal cancer patients receiving preoperative chemoradiotherapy, high *VSTM2L* expression was correlated with poor therapeutic response and adverse clinical outcomes (Liu et al., 2021). It was previously reported that *VSTM2L* was expressed at a low level and predicted poor prognosis in gastric cancer and rectal cancer (Hu et al., 2018; Liu et al., 2021), which was confirmed by our observations in STAD. Our results demonstrated that *VSTM2L* was significantly upregulated in 11 cancers and downregulated in 11 cancers. *VSTM2L* had different prognostic values across cancers: high expression was related to worse prognosis in BLCA, DLBC, GBM, KIRC, LUSC, MESO, OV, STAD, THCA, THYM, UCEC, and UVM but was associated with a good prognosis in KIRP, LGG, and PCPG. Further study is needed to clarify the reasons for the dual role of *VSTM2L* in cancers.

As vital components of the TIME, TIICs are a marker for prognosis and response to immunotherapy in multiple malignancies (Hendry et al., 2017; Zhang and Zhang, 2020). Many other markers have been examined for their utility in revealing susceptibility to immunotherapy including MSI, TMB, and immune checkpoint markers (Vrána et al., 2018; Chan et al., 2019; Li et al., 2020). Components of the IL-4 signaling pathway such as ALOX5, EGR1, SPAG1, NCF2, and ATXN1 are known to affect the immune response (Lee et al., 2009; Rajaraman et al., 2009; Siliņa et al., 2011; Li et al., 2019; Didonna et al., 2020; Wei et al., 2020); their regulation by *VSTM2L* induced chemoradiotherapy resistance in rectal cancer through downstream IL-4 signaling which subsequently affects the progress of cell proliferation and apoptosis (Liu et al., 2021). ALOX5 enhances colorectal cancer cell growth while EGR1 promotes colorectal cancer (Li et al., 2019; Wei et al., 2020; Liu et al., 2021). NCF2 regulates the antiapoptotic role of p53 in cancer cells exhibited by increased apoptosis due to loss of NCF2 and the associated upregulation of ATXN1 during VSTM2L overexpression suggested a link with cancer regulation (Lee et al., 2009; Rajaraman et al., 2009; Siliņa et al., 2011; Didonna et al., 2020). We, therefore, investigated the relationship between aberrant *VSTM2L* expression and TIICs, immunomodulators, TMB, MSI, and immune and stromal scores in different cancers, which has not been previously reported. Immunostimulators, immunoinhibitors, and MHC molecules with the top three highest correlation scores were STING1, CXCL12, VSIR, KDR, PVRL2, ADORA2A, HLA-DMA, HLA-DOB, and HLA-DPB1. We also found that *VSTM2L* was positively correlated with TMB in ACC and KIRP and negatively correlated with both TMB and MSI in COAD, STAD, and UCEC. It is worth noting that *VSTM2L* had prognostic value in high TMB STAD patients and low TMB KIRP patients; the opposite effects may imply that *VSTM2L* has distinct immunomodulatory functions in these cancers. The correlation between *VSTM2L* and the immune scores in different cancer suggest that aberrant *VSTM2L* expression may alter the TIME (Hendry et al., 2017). Thus, the regulation of VSTM2L modulates cancer development and progression. Therefore, the dysregulation of *VSTM2L* and immune markers among cancer patients suggest a crucial role of *VSTM2L* in the modulation of TIME (Binnewies et al., 2018; Hinshaw and Shevde, 2019). Taken together, the significant expression of tumor-infiltrating immune cells, immunostimulators, immunoinhibitors, major histocompatibility complex molecules, among others in various cancers point to an association between changes in VSTM2L and disease prognosis and development.

Our results indicate that *VSTM2L* is a promising independent prognostic factor in STAD and KIRP. A previous study has shown that *VSTM2L* was downregulated in *H. pylori*-positive gastric cancer patients compared to patients who were *H. pylori*-negative in TCGA and was expressed at a lower level in gastric cancer tissue compared to adjacent normal tissue (Hu et al., 2018), which was supported by our work. However, in the same study, the Kaplan-Meier analysis of 72 gastric cancer patients showed no association between *VSTM2L* expression level and patient survival based on TCGA data, which differed from our findings, possibly because of the different number of available TCGA samples that were analyzed (Hu et al., 2018). A negative correlation was found between *VSTM2L* and CIMP, and a CIMP-related gene signature comprising six genes (*VSTM2L*, CST6, SLC7A2, RAB3B, IGFBP1, and EVX2) stratified gastric cancer patients into high‐ and low-risk groups with distinct prognoses (Zeng et al., 2020). We confirmed the different expression patterns of *VSTM2L* in STAD and KIRP compared to normal tissue using TIMER, TCGA, GTEx, and HPA. Surprisingly, using TCGA, Kaplan–Meier Plotter, and GEO data we found that *VSTM2L* predicted opposite clinical outcomes in STAD and KIRP. Elevated *VSTM2L* expression was related to worse prognosis including OS, DSS, DFI, or PFI in STAD; on the contrary, it was positively correlated with longer survival in KIRP. The prognostic role of *VSTM2L* in KIRP has not been previously reported. We used datasets from multiple databases to perform a pan-cancer analysis of the effects of *VSTM2L* in cancer, but the main limitation in our study is the lack of experimental data to support our findings.

In summary, we showed that *VSTM2L* has distinct expression patterns, prognostic value, and relationship with the TIME of different cancers. Kaplan–Meier survival and Cox regression analyses revealed that upregulation of *VSTM2L* was associated with poor prognosis in STAD and good OS in KIRP. Our results also demonstrate for the first time that aberrant expression of *VSTM2L* was associated with the TIME including TIICs, immunostimulators, immunoinhibitors, MHC molecules, TMB, MSI, and immune and stromal scores in various cancers. These findings provide a basis for more in-depth investigations of *VSTM2L* function and interaction with the TIME, and suggest that *VSTM2L* is a potential target for cancer immunotherapy.

## Data Availability

Publicly available datasets were analyzed in this study. This data can be found here: https://xena.ucsc.edu/ https://commonfund.nih.gov/GTex https://portals.broadinstitute.org/ccle https://cistrome.shinyapps.io/timer/ http://cis.hku.hk/TISIDB/index.php http://kmplot.com/analysis/ https://www.ncbi.nlm.nih.gov/geo/.
