# Supplementary material for: Pan-Cancer Analysis Reveals the Multidimensional Expression and Prognostic and Immunologic Roles of VSTM2L in Cancer
Source: Front Mol Biosci. 2022 Jan 27;8:792154. doi: 10.3389/fmolb.2021.792154 (PMC8829123; doi:10.3389/fmolb.2021.792154)
Supplement: Supplementary file 1 [file DataSheet2.PDF]

Cancer	VTCN1 cor	VTCN1 adj.p	TIGIT cor	TIGIT adj.p	TGFBR1 cor	TGFBR1 adj.p	TGFB1 cor	TGFB1 adj.p	PVRL2 cor	PVRL2 adj.p	PDCD1LG2 cor	PDCD1LG2 adj.p
ACC	-0.08934	0.619483	-0.25857	0.057068	0.053238	0.787793	0.046933	0.736512	0.402434	0.000859	-0.20236	0.18423
BLCA	0.144037	0.01092	0.273907	1.87E-07	0.308651	3.74E-09	0.100861	0.083454	0.314144	8.53E-10	0.39991	1.69E-15
BRCA	0.079898	0.021987	0.180539	3.27E-08	0.158247	1.76E-06	0.14939	5.25E-06	-0.05328	0.147355	0.1761	4.10E-08
CESC	0.074313	0.408544	0.03538	0.801905	0.145192	0.036644	0.112045	0.095646	0.349034	2.27E-09	0.051542	0.567551
CHOL	-0.087	0.744096	0.40103	0.043861	0.202831	0.448489	0.133848	0.52899	0.035264	0.88233	0.517632	0.00544
COAD	-0.0295	0.68233	-0.00845	0.908179	0.192149	0.000199	0.143426	0.006434	-0.01989	0.813495	0.068846	0.291985
DLBC	0.183712	0.408544	0.07208	0.835148	-0.1206	0.571308	0.080221	0.65312	-0.00597	0.967874	-0.05341	0.798279
ESCA	0.039165	0.744096	0.222232	0.011817	0.033587	0.787793	0.013654	0.898581	0.147419	0.095229	0.107306	0.291985
GBM	0.084493	0.504676	0.001097	0.989259	0.152433	0.142479	-0.0041	0.968185	-0.0259	0.834045	-0.15282	0.158158
HNSC	0.206364	9.93E-06	0.097196	0.064085	0.061114	0.326491	0.100975	0.046742	0.195633	2.98E-05	0.03491	0.571489
KICH	0.151479	0.408544	0.271853	0.064085	-0.06381	0.787793	0.219374	0.122823	0.145164	0.349782	0.099281	0.571489
KIRC	0.221978	1.62E-06	0.113031	0.027716	0.06725	0.268826	0.112007	0.022717	0.099717	0.047352	0.054337	0.397971
KIRP	0.291067	2.59E-06	-0.18984	0.006631	-0.05257	0.532004	-0.20746	0.001504	0.321916	1.17E-07	-0.26552	2.60E-05
LGG	-0.13095	0.009603	-0.00763	0.908179	-0.3341	2.55E-13	-0.3269	1.03E-12	-0.23461	3.49E-07	-0.2792	1.56E-09
LIHC	0.180353	0.001751	-0.11284	0.066181	-0.07746	0.287201	0.075301	0.203777	0.068124	0.304718	-0.05876	0.450292
LUAD	0.228255	1.62E-06	-0.04811	0.460657	-0.12191	0.022415	0.199577	2.87E-05	0.275432	1.63E-09	-0.04739	0.452922
LUSC	-0.0829	0.149931	0.253079	1.23E-07	-0.04335	0.493192	0.181778	0.000213	0.386836	9.96E-18	0.127108	0.014596
MESO	-0.02019	0.871155	0.063589	0.801905	0.450627	7.92E-05	0.496993	6.50E-06	0.112689	0.412015	0.160786	0.291985
OV	0.021731	0.807266	-0.01191	0.908179	0.11419	0.125428	0.281123	5.25E-06	0.201285	0.001301	0.031884	0.682791
PAAD	-0.02359	0.837745	-0.09485	0.375633	0.042213	0.766334	0.197646	0.020003	0.281874	0.000528	-0.04846	0.631693
PCPG	-0.19582	0.021987	0.02552	0.888053	0.182861	0.042287	-0.23249	0.005454	0.011098	0.904738	-0.22054	0.01037
PRAD	-0.16833	0.000642	-0.01837	0.853145	-0.00669	0.974217	-0.06718	0.199043	0.291597	4.31E-10	-0.11459	0.029971
READ	-0.05845	0.626834	0.163962	0.073233	0.079605	0.473791	0.146573	0.099188	0.062182	0.549809	0.059947	0.57155
SARC	-0.04275	0.656737	-0.16812	0.025935	0.067982	0.457939	-0.12641	0.083454	0.077228	0.330096	-0.28254	2.45E-05
SKCM	-0.05643	0.408544	0.005996	0.919739	-0.04908	0.460403	0.088083	0.097565	0.076302	0.178433	-0.02963	0.631693
STAD	0.250173	1.62E-06	0.069229	0.303252	0.24453	4.58E-06	0.320521	4.55E-10	0.065967	0.299724	0.077223	0.273501
TGCT	0.584337	8.33E-14	-0.36831	2.35E-05	-0.00198	0.981341	0.294216	0.001145	0.512533	4.03E-10	-0.342	9.20E-05
THCA	0.542828	9.62E-39	0.280083	5.02E-09	0.118806	0.026508	0.06248	0.212359	-0.12508	0.011088	0.280537	1.56E-09
THYM	0.078318	0.607974	0.028801	0.888053	-0.18712	0.116328	-0.05821	0.619123	-0.25682	0.011088	0.273463	0.010041
UCEC	-0.01813	0.791586	-0.01961	0.835837	0.005333	0.974217	0.149245	0.001719	0.154502	0.000981	0.011798	0.846991
UCS	-0.22757	0.197028	-0.07856	0.801905	0.366671	0.022335	-0.08251	0.619123	-0.03221	0.877806	-0.11194	0.571489
UVM	-0.02564	0.871155	0.295533	0.025935	-0.02274	0.961514	0.21737	0.095935	0.060338	0.743696	-0.08973	0.571489

Cancer	PDCD1 cor	PDCD1 adj.p	LGALS9 cor	LGALS9 adj.p	LAG3 cor	LAG3 adj.p	KIR2DL3 cor	KIR2DL3 adj.p	KIR2DL1 cor	KIR2DL1 adj.p	KDR cor	KDR adj.p
ACC	-0.16361	0.352104	-0.11887	0.42397	-0.02111	0.92706	-0.1275	0.500567	-0.1145	0.586296	-0.22468	0.080888
BLCA	0.297613	1.15E-08	0.119231	0.045632	0.322307	1.03E-09	0.226315	0.000155	0.185955	0.002111	0.010631	0.897815
BRCA	0.19749	7.80E-10	0.18527	7.95E-09	0.155596	2.86E-06	0.05808	0.180454	0.056443	0.20431	0.027048	0.510988
CESC	0.079387	0.366056	0.029943	0.752293	0.041596	0.595426	0.038377	0.774784	0.01378	0.953268	0.143955	0.023403
CHOL	0.249678	0.352104	-0.06718	0.844911	0.210296	0.349275	0.248897	0.366934	-0.02269	0.968098	0.369112	0.048605
COAD	-0.05645	0.396347	-0.1018	0.073452	-0.08016	0.192479	-0.10356	0.118602	-0.09047	0.192801	0.268608	3.47E-08
DLBC	-0.10117	0.705476	0.091294	0.716206	0.015306	0.941293	0.254303	0.231771	0.157473	0.586296	-0.08055	0.690996
ESCA	0.189403	0.035708	0.120409	0.170929	0.135946	0.162553	0.012712	0.914344	-0.01258	0.968098	0.205335	0.01063
GBM	0.13107	0.283541	0.001414	0.98616	-0.00893	0.941293	0.007303	0.928612	0.00326	0.968098	-0.03243	0.768552
HNSC	0.057396	0.366056	0.190634	7.72E-05	0.041405	0.493002	0.010482	0.914344	0.027026	0.887109	0.211655	4.73E-06
KICH	0.223016	0.205356	0.255216	0.090883	0.32402	0.02447	0.250345	0.155002	0.055544	0.939703	-0.10909	0.510988
KIRC	0.14348	0.00447	0.186041	8.80E-05	0.135789	0.007454	-0.03067	0.76771	-0.09199	0.141286	-0.29856	1.56E-11
KIRP	-0.01524	0.837927	0.105344	0.133207	-0.15851	0.022778	-0.15383	0.069534	-0.099	0.264043	-0.44792	1.02E-14
LGG	-0.24441	1.49E-07	-0.35076	8.80E-15	-0.2156	6.13E-06	-0.05168	0.500567	0.002045	0.968098	-0.17712	0.000174
LIHC	-0.09679	0.192473	0.037878	0.64413	-0.07661	0.279778	-0.00822	0.914344	0.018093	0.941013	0.163543	0.004197
LUAD	0.025753	0.735536	0.116784	0.026601	-0.06506	0.279778	-0.00849	0.914344	-0.0134	0.941013	0.252104	3.76E-08
LUSC	0.324624	3.72E-12	0.261982	2.62E-08	0.220839	5.96E-06	0.105409	0.109078	0.135293	0.02408	0.355726	4.33E-15
MESO	0.280528	0.033972	0.023019	0.924885	-0.15475	0.279778	-0.40694	0.001835	-0.2689	0.078592	-0.33781	0.003927
OV	-0.01602	0.837927	-0.01378	0.924885	-0.07101	0.349275	0.055756	0.606191	0.05712	0.586296	0.276396	4.73E-06
PAAD	-0.03614	0.76486	0.003285	0.98616	-0.05695	0.595426	0.084657	0.500567	-0.12972	0.256911	-0.0863	0.385686
PCPG	-0.04751	0.724663	-0.17601	0.047414	0.207684	0.018274	-0.01671	0.914344	0.022073	0.941013	-0.21971	0.007401
PRAD	-0.04911	0.438401	-0.08664	0.112263	-0.13312	0.011663	-0.0257	0.800288	-0.09984	0.129416	-0.21349	6.10E-06
READ	0.104785	0.366056	0.007874	0.968209	0.055655	0.595426	0.113114	0.366934	0.119973	0.329732	0.009622	0.949561
SARC	0.032627	0.750613	-0.10989	0.133816	-0.03616	0.68072	-0.13888	0.118602	-0.1452	0.10948	0.033809	0.690996
SKCM	0.015167	0.837927	0.057466	0.323586	0.008294	0.92706	-0.02308	0.823028	0.069214	0.33408	0.030758	0.631829
STAD	0.027961	0.735536	-0.15912	0.005083	-0.05023	0.464982	-0.10551	0.126534	-0.12591	0.078592	0.177279	0.000874
TGCT	-0.41936	6.15E-07	-0.15089	0.124394	-0.42732	9.87E-07	-0.3043	0.002042	-0.30603	0.002111	0.59696	9.99E-15
THCA	0.055717	0.380924	0.29663	1.70E-10	0.207705	1.53E-05	-0.05923	0.428537	-0.16817	0.002111	-0.43174	6.33E-23
THYM	-0.01386	0.903191	-0.02205	0.924885	-0.03327	0.841817	-0.26985	0.028771	-0.05449	0.887109	-0.16772	0.111823
UCEC	0.037311	0.569874	-0.0528	0.323586	0.036275	0.548974	-0.01043	0.914344	0.056247	0.446636	0.122371	0.009385
UCS	0.041289	0.837927	-0.26303	0.106813	-0.24008	0.169515	-0.07481	0.800288	0.05389	0.941013	0.006028	0.979098
UVM	0.379568	0.002947	0.300609	0.024512	0.4485	0.000172	0.137616	0.496637	0.052049	0.939703	0.325715	0.007519

Cancer	IL10RB cor	IL10RB adj.p	IL10 cor	IL10 adj.p	IDO1 cor	IDO1 adj.p	HAVCR2 cor	HAVCR2 adj.p	CTLA4 cor	CTLA4 adj.p	CSF1R cor	CSF1R adj.p
ACC	0.172201	0.224585	-0.1082	0.456728	0.220204	0.127931	-0.12451	0.476964	-0.23421	0.083896	-0.24048	0.072835
BLCA	0.086029	0.183121	0.420317	2.71E-17	0.192298	0.000618	0.356629	4.44E-12	0.272558	1.77E-07	0.33891	4.02E-11
BRCA	0.137197	6.60E-05	0.167149	2.45E-07	0.145661	1.23E-05	0.116856	0.00082	0.202394	2.48E-10	0.09017	0.01104
CESC	0.129494	0.078275	0.056825	0.456728	-0.03294	0.685964	-0.03044	0.721422	0.068246	0.406821	-0.00445	0.962255
CHOL	-0.01493	0.94847	0.260763	0.265907	0.279022	0.203447	0.435521	0.039666	0.395624	0.052077	0.366281	0.065912
COAD	0.109377	0.069859	0.058786	0.334741	-0.06154	0.301749	0.023682	0.721422	0.004021	0.955499	0.199608	0.000132
DLBC	0.11713	0.552089	0.000868	0.995326	-0.03713	0.85962	-0.13287	0.525668	0.051997	0.829252	0.096939	0.660871
ESCA	0.029426	0.789615	0.148508	0.116398	0.118953	0.203447	0.103059	0.309948	0.181219	0.048129	0.091151	0.377775
GBM	-0.1288	0.214406	0.012843	0.920844	-0.14136	0.191388	0.003679	0.964001	0.10245	0.39146	0.029006	0.825028
HNSC	0.192384	9.59E-05	0.147017	0.004197	-0.02286	0.708518	0.111867	0.041796	0.153037	0.002572	0.171117	0.000487
KICH	-0.10446	0.552089	0.222823	0.169709	-0.1422	0.364154	0.118548	0.525668	0.234317	0.122667	0.216932	0.160379
KIRC	-0.074	0.183121	0.128371	0.01195	-0.14829	0.002839	-0.1094	0.041796	0.091622	0.083635	0.120263	0.019763
KIRP	0.142523	0.060561	-0.10762	0.168099	-0.32985	1.74E-07	-0.1061	0.203459	-0.03307	0.718585	0.05833	0.495751
LGG	-0.42393	2.55E-22	-0.28376	6.91E-10	-0.18755	0.000144	-0.30889	9.59E-12	-0.03741	0.546726	-0.23656	7.18E-07
LIHC	0.003367	0.94847	-0.00949	0.920844	-0.1154	0.07497	-0.03458	0.698831	-0.12691	0.048129	0.007775	0.927746
LUAD	0.119223	0.038599	-0.01936	0.777868	0.004178	0.924652	0.04206	0.525668	-0.0205	0.762662	0.061664	0.309183
LUSC	0.069105	0.222556	0.309222	2.92E-11	0.254195	1.74E-07	0.316468	8.15E-12	0.292448	3.27E-10	0.402483	2.49E-19
MESO	0.026974	0.893482	-0.13644	0.334741	0.080174	0.5765	0.121273	0.476964	0.120362	0.426879	0.001494	0.989039
OV	0.122113	0.102285	0.183623	0.00589	-0.09304	0.203447	0.147721	0.041796	-0.01057	0.949574	0.242487	0.000132
PAAD	0.08477	0.370319	-0.10846	0.282808	0.018116	0.85962	-0.041	0.721422	-0.07589	0.481019	-0.0768	0.490961
PCPG	-0.18074	0.060561	-0.07222	0.456728	-0.19546	0.030418	-0.11166	0.269051	-0.04229	0.718585	-0.07006	0.516493
PRAD	0.061208	0.276227	-0.06484	0.282808	0.065115	0.255273	-0.0441	0.525668	0.020491	0.762662	-0.10319	0.053174
READ	0.131378	0.183121	0.043233	0.703298	0.082375	0.401901	0.133823	0.22835	0.011639	0.953177	0.188982	0.045
SARC	0.078423	0.319278	-0.09506	0.265907	-0.08608	0.277317	-0.0958	0.259689	-0.09445	0.2575	-0.0563	0.522792
SKCM	0.029787	0.629105	-0.05902	0.334741	0.034034	0.5765	-0.00424	0.950567	-0.04126	0.530928	0.031915	0.65277
STAD	0.022055	0.769594	0.108112	0.085076	-0.12093	0.042136	0.030927	0.706429	-0.06095	0.39146	0.229061	2.42E-05
TGCT	0.389004	1.73E-05	-0.29219	0.001903	-0.38651	1.23E-05	-0.30516	0.000976	-0.42859	2.97E-07	0.022755	0.854681
THCA	0.087699	0.12795	0.14757	0.004197	0.150006	0.002839	0.241757	3.33E-07	0.348879	2.06E-14	0.134897	0.010174
THYM	-0.19185	0.102285	-0.0166	0.920844	-0.10705	0.364154	0.02214	0.87601	0.10341	0.426879	-0.02449	0.854681
UCEC	0.07592	0.180202	-0.00422	0.945297	-0.04885	0.364154	-0.01315	0.867908	-0.09007	0.083635	0.057088	0.333214
UCS	0.107726	0.552089	-0.02398	0.920844	-0.2618	0.127931	0.022492	0.913794	0.004797	0.971752	0.067086	0.775022
UVM	0.271285	0.060561	0.110444	0.456728	0.370788	0.002839	0.236123	0.107622	0.256473	0.061862	0.265776	0.045819

Cancer	CD274 cor	CD274 adj.p	CD244 cor	CD244 adj.p	CD160 cor	CD160 adj.p	CD96 cor	CD96 adj.p	BTLA cor	BTLA adj.p	ADORA2A cor	ADORA2A adj.p
ACC	-0.16931	0.271583	-0.17491	0.253948	-0.07115	0.687991	-0.07629	0.636236	0.234087	0.079701	0.069596	0.637904
BLCA	0.252339	3.20E-06	0.291955	2.47E-08	0.22668	3.00E-05	0.025915	0.687669	0.268837	3.48E-07	0.354477	3.19E-12
BRCA	0.100965	0.004562	0.176505	3.78E-08	0.045336	0.25318	0.177151	1.03E-07	0.180442	3.33E-08	0.038165	0.284049
CESC	-0.09133	0.260831	0.004606	0.978618	0.09064	0.227155	0.059227	0.431054	0.010817	0.887402	0.170231	0.008037
CHOL	0.389189	0.062402	0.447619	0.035386	-0.05405	0.833254	0.373745	0.070693	0.471936	0.011268	0.492149	0.007068
COAD	-0.10843	0.062402	-0.08439	0.177934	0.03748	0.564793	-0.03094	0.636236	0.100735	0.069176	0.159335	0.002256
DLBC	-0.07501	0.741425	0.025185	0.950844	0.200065	0.300455	0.15393	0.431054	0.052323	0.827366	-0.15143	0.392532
ESCA	0.068453	0.567252	0.157059	0.099923	0.293571	0.000286	0.21274	0.024302	0.268185	0.000993	0.286593	0.000309
GBM	-0.14276	0.195872	0.234919	0.023114	0.370287	2.46E-05	-0.08891	0.431054	-0.05922	0.583908	0.250482	0.005971
HNSC	-0.03761	0.598024	0.00665	0.950844	0.191198	7.27E-05	0.099276	0.070693	0.176422	0.000253	0.242004	2.15E-07
KICH	-0.16034	0.345083	0.153013	0.399972	0.26557	0.089002	0.30112	0.062288	0.294264	0.041178	0.169337	0.261049
KIRC	-0.11457	0.032423	0.008089	0.950844	0.01265	0.833254	0.130629	0.020111	0.155145	0.001297	-0.20116	1.90E-05
KIRP	-0.38224	6.39E-10	0.024655	0.901149	0.287404	1.28E-05	-0.06224	0.431054	0.093237	0.22622	-0.12002	0.086544
LGG	0.013532	0.820661	0.334394	2.41E-13	0.271184	1.51E-08	-0.12529	0.024959	-0.24416	2.57E-07	0.019142	0.73825
LIHC	0.034665	0.697427	-0.05494	0.506461	-0.16806	0.004627	-0.09223	0.168945	-0.07016	0.284031	0.0434	0.50569
LUAD	0.094201	0.093063	-0.09294	0.099923	-0.06461	0.26023	-0.05813	0.357759	-0.04979	0.370587	4.20E-05	0.999242
LUSC	0.025508	0.734123	0.268872	1.92E-08	0.211112	2.46E-05	0.257274	1.03E-07	0.293157	8.75E-10	0.447172	2.12E-24
MESO	0.017588	0.8939	-0.00585	0.978618	-0.1987	0.146488	-0.00906	0.936876	0.03472	0.832814	0.284519	0.018913
OV	-0.02572	0.749312	0.026607	0.889061	0.006849	0.928705	0.0215	0.78815	-0.07995	0.275175	0.077904	0.261049
PAAD	0.09987	0.333586	-0.14418	0.144426	0.025939	0.833254	-0.08097	0.431054	-0.04875	0.626657	-0.05632	0.550237
PCPG	0.182324	0.051009	-0.05845	0.643628	0.023591	0.833254	0.013583	0.901035	0.012704	0.887402	-0.18	0.036044
PRAD	-0.14352	0.006603	-0.10931	0.053194	-0.15873	0.001884	-0.08293	0.151607	-0.00428	0.924162	-0.12687	0.012199
READ	-0.03764	0.741425	-0.01472	0.950844	-0.00201	0.979527	0.11192	0.318133	0.098736	0.31639	0.103635	0.262762
SARC	-0.28755	2.43E-05	-0.10828	0.191533	-0.0515	0.563067	-0.07347	0.431054	-0.09383	0.238687	-0.00057	0.999242
SKCM	-0.01077	0.85852	0.041727	0.569524	0.046678	0.462332	-0.0381	0.564641	0.016987	0.827366	0.05171	0.350286
STAD	-0.17567	0.002155	0.0441	0.569524	0.133865	0.022953	0.092592	0.14876	0.159718	0.003982	0.272674	2.15E-07
TGCT	-0.418	2.05E-06	-0.21509	0.036487	-0.21518	0.027275	-0.37304	2.58E-05	-0.35762	4.69E-05	-0.11954	0.23218
THCA	0.19762	5.65E-05	0.118151	0.036487	-0.08339	0.146488	0.104746	0.065763	0.195797	4.91E-05	-0.17879	0.000249
THYM	0.138746	0.271583	0.223654	0.053194	0.110216	0.38462	-0.00731	0.936876	0.212424	0.046691	-0.00658	0.992742
UCEC	-0.01719	0.765428	-0.00902	0.950844	0.036907	0.5569	0.025353	0.672455	-0.03574	0.522577	0.219185	1.89E-06
UCS	-0.26641	0.12043	0.00363	0.978618	-0.14176	0.450573	-0.14739	0.431054	-0.12179	0.498054	0.220443	0.180724
UVM	0.109025	0.559519	0.02255	0.950844	0.123114	0.442568	0.211626	0.14876	0.160951	0.267473	0.248125	0.05883
