# Supplementary material for: Pan-Cancer Analysis Reveals the Multidimensional Expression and Prognostic and Immunologic Roles of VSTM2L in Cancer
Source: Front Mol Biosci. 2022 Jan 27;8:792154. doi: 10.3389/fmolb.2021.792154 (PMC8829123; doi:10.3389/fmolb.2021.792154)
Supplement: Supplementary file 3 [file DataSheet4.PDF]

CancerType	ImmuneScore *P*	ImmuneScore r	StromalScore *P*	StromalScore r
ACC	0.018955238	-0.26	0.026909033	-0.25
BLCA	1.58E-09	0.29	6.44E-14	0.36
BRCA	9.40E-13	0.21	4.35E-12	0.21
CESC	0.456727063	0.043	0.002533773	0.17
CHOL	0.008357477	0.44	0.015752148	0.4
COAD	0.841034514	-0.0093	2.43E-05	0.19
DLBC	0.827988207	-0.032	0.358591714	-0.14
ESCA	0.041783591	0.16	0.006033375	0.22
GBM	0.371823411	-0.069	0.702834047	0.03
HNSC	0.326743963	0.044	0.01640931	0.11
KICH	0.077708838	0.22	0.106578897	0.2
KIRC	6.51E-05	0.17	4.60E-05	0.18
KIRP	0.197858685	-0.076	0.00222279	-0.18
LAML	0.064270282	-0.15	0.125125989	-0.13
LGG	2.20E-16	-0.4	3.30E-09	-0.25
LIHC	0.204619724	-0.066	0.008148487	0.14
LUAD	0.985183569	0.00081	0.49896949	0.03
LUSC	2.20E-16	0.39	1.80E-13	0.32
MESO	0.551227606	0.065	0.002844792	0.32
OV	0.928105819	-0.0046	0.000146253	0.19
PAAD	0.024667291	-0.17	0.011605585	-0.19
PCPG	0.008938448	-0.19	1.98E-06	-0.35
PRAD	0.051779921	-0.087	0.000386332	-0.16
READ	0.313641277	0.078	0.021646809	0.18
SARC	0.187875871	-0.081	0.917667611	-0.0064
SKCM	0.50529613	0.031	0.928763745	0.0041
STAD	0.075315756	0.092	7.56E-10	0.31
TGCT	1.54E-06	-0.38	2.20E-16	0.55
THCA	6.65E-14	0.33	0.000130091	0.17
THYM	0.002000974	0.28	0.385592993	-0.08
UCEC	0.554278691	0.025	0.000648678	0.15
UCS	0.058086988	-0.26	0.478438156	0.096
UVM	0.024576037	0.25	0.04995544	0.22
