# Supplementary material for: Pan-Cancer Analysis Reveals the Multidimensional Expression and Prognostic and Immunologic Roles of VSTM2L in Cancer
Source: Front Mol Biosci. 2022 Jan 27;8:792154. doi: 10.3389/fmolb.2021.792154 (PMC8829123; doi:10.3389/fmolb.2021.792154)
Supplement: Supplementary file 6 [file DataSheet3.PDF]

Cancer	B2M cor	B2M adj.p	HLA-A cor	HLA-A adj.p	HLA-B cor	HLA-B adj.p	HLA-C cor	HLA-C adj.p	HLA-DMA cor	HLA-DMA adj.p
ACC	-0.1998783	0.193444	-0.187999	0.1915939	-0.1003408	0.5413386	-0.0652629	0.6971063	-0.1554284	0.3115978
BLCA	0.1209557	0.0525269	0.1107874	0.0741235	0.1768047	0.0016621	0.1435693	0.0182965	0.2149074	7.95E-05
BRCA	0.0881485	0.0152635	0.1124657	0.0012381	0.1179873	0.0005843	0.0961868	0.0080198	0.1862276	4.85E-09
CESC	-0.0246719	0.7850372	0.0533588	0.5475131	0.0466989	0.5733055	0.1105297	0.1424601	0.0696459	0.3591107
CHOL	0.3127413	0.1687859	0.23861	0.2928597	0.3068211	0.1566029	0.197426	0.4338658	0.3127413	0.1406549
COAD	-0.1490284	0.0092093	-0.1371529	0.0131632	-0.0966722	0.1188726	-0.0981663	0.1098852	-0.0514578	0.4026428
DLBC	-0.2120061	0.3115944	-0.0174772	0.9354459	-0.2361051	0.1846628	-0.2116804	0.2972913	-0.2547764	0.1695507
ESCA	0.0470225	0.7435789	0.0522156	0.6843273	0.0963743	0.2952265	0.0414749	0.6971063	0.1657576	0.0877833
GBM	-0.2392713	0.0148088	-0.2574987	0.0065604	-0.2046594	0.0446391	-0.1481243	0.1592	-0.0504068	0.6306622
HNSC	0.0540655	0.3782682	0.0822904	0.1506814	0.098728	0.0802819	0.0820361	0.1526815	0.1144993	0.0392697
KICH	0.0806605	0.7435789	0.0058867	0.9625846	0.0934568	0.6072541	-0.1407592	0.4338658	0.1791481	0.2858549
KIRC	-0.1218046	0.0194522	-0.0964661	0.0741235	-0.1552	0.0016621	-0.1434053	0.0059975	-0.0272193	0.6306622
KIRP	-0.1416786	0.0525269	-0.0349561	0.6915676	-0.0113755	0.8916285	-0.0110852	0.9293734	-0.125911	0.0879295
LGG	-0.2991034	1.60E-10	-0.2801389	1.24E-09	-0.2791877	1.44E-09	-0.2246214	2.52E-06	-0.345144	1.40E-14
LIHC	0.065321	0.3782682	-0.0573377	0.4510729	-0.0254381	0.7465766	0.0102274	0.9293734	-0.0211394	0.7403855
LUAD	-0.0215401	0.7638391	0.0735988	0.1915939	0.0722133	0.1846628	0.0477334	0.4338658	0.0503122	0.3913932
LUSC	0.257244	6.87E-08	0.3265858	1.29E-12	0.33415	6.24E-13	0.3144267	1.17E-11	0.4171626	6.51E-21
MESO	-0.1597653	0.3097058	-0.190694	0.1808392	-0.1948859	0.1566029	-0.186083	0.1875711	0.049045	0.7243398
OV	-0.0288023	0.7638391	-0.0466885	0.6193545	0.0228954	0.7901827	-0.0101984	0.9293734	-0.0465461	0.5412729
PAAD	0.0682527	0.5823571	0.0401	0.7200764	-0.0359948	0.7465766	0.0029691	0.9851544	-0.095106	0.3422895
PCPG	-0.036027	0.7638391	-0.1740493	0.0637158	-0.1154514	0.2028409	-0.0987331	0.3543847	-0.1749884	0.0738666
PRAD	-0.0141189	0.8369924	0.0290803	0.6855191	0.0760741	0.1798263	0.1161894	0.0420219	-0.0349566	0.5454418
READ	-0.088325	0.4296696	-0.0889494	0.4425028	-0.0892616	0.3744729	-0.0046488	0.9851544	-0.0148946	0.8489452
SARC	-0.2368984	0.0009202	-0.287411	2.46E-05	-0.2540111	0.0002727	-0.3172544	2.30E-06	-0.1177118	0.1365336
SKCM	0.0151382	0.8369924	-0.0119865	0.9354459	0.0748933	0.1846628	0.0400206	0.5516678	0.0387904	0.5345975
STAD	-0.0626948	0.3782682	-0.0065712	0.9354459	0.0136292	0.857207	-0.028634	0.6971063	-0.0124856	0.8217919
TGCT	0.0015556	0.9849264	0.0139757	0.9354459	-0.0216134	0.857207	0.0886244	0.4338658	-0.1742337	0.0879295
THCA	0.2940135	2.63E-10	0.3510659	1.32E-14	0.3223021	1.82E-12	0.322767	3.34E-12	0.345049	1.49E-14
THYM	-0.0692895	0.6954738	0.0101882	0.9354459	0.0462324	0.7465766	0.0360303	0.8188566	0.0452948	0.7122945
UCEC	-0.1270681	0.0148088	-0.1422709	0.0049505	-0.0736457	0.1798263	-0.0626422	0.2972913	-0.1639143	0.0006921
UCS	-0.3085299	0.0601296	-0.2616671	0.1314072	-0.309567	0.0695087	-0.3142987	0.0690601	-0.2895385	0.0879295
UVM	0.5016643	2.13E-05	0.4224566	0.0007585	0.4535631	0.0002387	0.4010783	0.0018156	0.3426395	0.009317

Cancer	HLA-DMB cor	HLA-DMB adj.p	HLA-DOA cor	HLA-DOA adj.p	HLA-DOB cor	HLA-DOB adj.p	HLA-DPA1 cor	HLA-DPA1 adj.p	HLA-DPB1 cor	HLA-DPB1 adj.p
ACC	0.0373661	0.7828578	-0.2557205	0.0655093	-0.3254325	0.0114262	-0.3093963	0.0246858	-0.2113194	0.1641488
BLCA	0.3018149	6.49E-09	0.2900748	3.16E-08	0.3200658	1.10E-09	0.297016	1.04E-08	0.3259276	2.99E-10
BRCA	0.1563514	1.87E-06	0.1576549	1.18E-06	0.1864236	6.19E-09	0.1330432	7.64E-05	0.1814289	1.08E-08
CESC	0.1079067	0.1390542	0.0318952	0.7010301	0.0540499	0.4772908	0.0210894	0.7925413	0.0361816	0.6215815
CHOL	0.3693694	0.0760532	0.4615187	0.0153582	0.3616474	0.0636109	0.4545689	0.0246858	0.5003861	0.0094206
COAD	-0.0387453	0.5943651	0.0941681	0.1099567	-0.0100246	0.8518466	0.0255082	0.7104251	0.0673307	0.277779
DLBC	-0.3294616	0.0683554	-0.2107034	0.3011455	-0.1699957	0.3674501	-0.2964611	0.1086703	-0.2561876	0.1918089
ESCA	0.1975266	0.0234595	0.2430651	0.0042838	0.2318032	0.0066628	0.1470566	0.1144267	0.1679258	0.081179
GBM	-0.0023588	0.9769141	-0.0108694	0.8939177	0.0239771	0.8090661	-0.083789	0.4663675	-0.0465033	0.649293
HNSC	0.1431615	0.0046149	0.151976	0.0028209	0.1452513	0.0043686	0.116399	0.0310634	0.1273075	0.0158832
KICH	0.1881452	0.2272296	0.1079231	0.5753916	0.112599	0.4796496	0.1510297	0.4043455	0.1534094	0.3365804
KIRC	0.004199	0.9466182	-0.0109427	0.8215445	0.043202	0.4564063	-0.0201776	0.7359105	0.007334	0.888058
KIRP	-0.0944205	0.2134612	-0.0889452	0.2752709	-0.1336565	0.057043	-0.1264409	0.0964722	-0.0444429	0.5636066
LGG	-0.3341985	2.51E-13	-0.2564129	3.43E-08	-0.2202114	2.91E-06	-0.2645216	1.04E-08	-0.2636648	1.08E-08
LIHC	-0.021482	0.7351657	-0.0196579	0.7843094	-0.1130303	0.0636109	0.0047043	0.9518396	-0.0007652	0.9882806
LUAD	0.0292563	0.5943651	0.0323556	0.6183431	-0.0178834	0.7617352	0.0268179	0.6796276	0.0446244	0.4305454
LUSC	0.33101	5.66E-13	0.4264169	5.96E-22	0.2500284	1.41E-07	0.3808049	3.92E-17	0.4084911	5.73E-20
MESO	0.0718269	0.5943651	0.107385	0.5154788	0.0969418	0.4796496	0.1020631	0.5138861	0.1440366	0.3053126
OV	-0.1720396	0.009666	-0.045491	0.5932642	-0.071623	0.3420835	-0.0024515	0.9661025	0.0212307	0.7706136
PAAD	-0.1134057	0.2272296	-0.0718641	0.5216677	-0.0442743	0.6741875	-0.1136631	0.2878744	-0.1303685	0.1918089
PCPG	-0.1505575	0.1148458	-0.0295833	0.7843094	0.0139781	0.8518466	0.010222	0.9382818	-0.0784449	0.4305454
PRAD	0.0294009	0.5943651	-0.1237238	0.0175308	-0.0140358	0.8090661	-0.0603532	0.340436	-0.076938	0.1918089
READ	0.0545586	0.5943651	0.2243351	0.01466	0.0430023	0.68499	0.1590637	0.1086703	0.2033082	0.0344418
SARC	-0.0987786	0.2134612	-0.0415435	0.6513769	-0.1828895	0.0111941	-0.1127912	0.1633189	-0.0943475	0.25835
SKCM	0.0297113	0.5943651	0.0176657	0.7843094	0.0237422	0.6940269	0.0400955	0.5314197	0.0574176	0.3365804
STAD	0.0370876	0.5943651	0.1392023	0.0153582	0.1797048	0.0013337	0.0587351	0.4043455	0.1007339	0.1341792
TGCT	-0.2573252	0.005913	-0.0968523	0.4334189	-0.2401831	0.0111941	-0.1873417	0.072324	-0.0724139	0.4884154
THCA	0.2069756	1.42E-05	0.2851934	1.11E-09	0.2852703	1.10E-09	0.3058412	3.51E-11	0.285904	6.61E-10
THYM	-0.1705327	0.1390542	-0.0938051	0.5136106	0.159518	0.1558202	-0.0615182	0.6726236	0.0622404	0.6054146
UCEC	-0.2164285	2.69E-06	0.0544773	0.3888625	-0.0616725	0.2508077	-0.0478851	0.4381081	-0.0440712	0.4305454
UCS	-0.1044205	0.5943651	0.0369458	0.8215445	-0.2160717	0.1935689	-0.0625486	0.7359105	-0.13119	0.4409152
UVM	0.1567276	0.2750517	0.2163619	0.1268227	0.2616619	0.0508005	0.2811299	0.0419412	0.2131271	0.1641488

Cancer	HLA-DQA1 cor	HLA-DQA1 adj.p	HLA-DQA2 cor	HLA-DQA2 adj.p	HLA-DQB1 cor	HLA-DQB1 adj.p	HLA-DRA cor	HLA-DRA adj.p	HLA-DRB1 cor	HLA-DRB1 adj.p
ACC	-0.2184518	0.1415947	-0.2599805	0.0689167	-0.20185	0.1985077	-0.2016796	0.1659792	-0.1934275	0.2062081
BLCA	0.3062384	5.26E-09	0.3047655	6.46E-09	0.2621911	9.29E-07	0.2864211	3.83E-08	0.2763478	1.38E-07
BRCA	0.1229572	0.0003467	0.1349685	5.65E-05	0.16015	9.29E-07	0.1618305	5.43E-07	0.1565671	1.44E-06
CESC	0.0324357	0.7034461	0.021482	0.7454636	0.025261	0.7200245	0.0294039	0.7157602	0.0241032	0.7708664
CHOL	0.3899614	0.0623884	0.2638353	0.2481207	0.553668	0.0026297	0.4954955	0.0121184	0.4607465	0.0208234
COAD	0.0498188	0.4597873	0.0952812	0.1107535	-0.0085289	0.8555583	-0.0335888	0.6270036	-0.0235052	0.7574382
DLBC	-0.3056882	0.0991276	-0.3345636	0.0689167	-0.2075554	0.3303316	-0.3340208	0.0681567	-0.306231	0.1054528
ESCA	0.0701281	0.507926	0.1109814	0.252571	0.0549259	0.6527379	0.1652894	0.0755348	0.1016906	0.3368141
GBM	-0.0060345	0.9819047	0.0210185	0.7964993	-0.0351715	0.7200245	-0.05555	0.6270036	-0.0144144	0.9196035
HNSC	0.1186549	0.0295413	0.1463884	0.0039732	0.0940673	0.1266011	0.1114528	0.0433076	0.1025628	0.0636192
KICH	0.2106274	0.1886154	0.1364799	0.439237	0.1811312	0.32342	0.1769562	0.2956332	0.220877	0.1992398
KIRC	0.0091883	0.8998837	0.019234	0.7110596	-0.0831968	0.1568932	-0.0047614	0.9607089	-0.0212256	0.7574382
KIRP	-0.1022403	0.1826406	-0.0792905	0.323859	-0.055366	0.55596	-0.1142636	0.1297971	-0.1521446	0.0344105
LGG	-0.2510092	9.91E-08	-0.2221046	3.45E-06	-0.1931655	7.94E-05	-0.2782916	1.66E-09	-0.2695806	6.44E-09
LIHC	-0.0394153	0.598794	-0.0299234	0.6654072	-0.036289	0.6527379	-0.0014723	0.980495	0.0026368	0.9596314
LUAD	0.0231085	0.7034461	0.067857	0.2481207	0.0902334	0.1269975	0.0343713	0.6233813	0.0351337	0.5683388
LUSC	0.2405563	5.00E-07	0.3391306	2.39E-13	0.2792114	4.01E-09	0.3624129	2.15E-15	0.3615248	1.30E-15
MESO	0.1480644	0.3259303	0.1053984	0.4907539	0.1281621	0.430553	0.1136728	0.4779362	0.0811402	0.5870916
OV	0.0293833	0.7034461	0.0957431	0.2137943	0.0267161	0.7200245	-0.0167819	0.8567799	-0.0549541	0.5043172
PAAD	-0.0864792	0.416176	-0.0361768	0.7007478	-0.0237169	0.771961	-0.108654	0.2953477	-0.0778786	0.5007897
PCPG	-0.0729828	0.5059606	0.065584	0.524679	-0.0516868	0.6527379	-0.1206889	0.2222775	-0.2068444	0.0208367
PRAD	-0.0661047	0.2814532	-0.0327278	0.5937476	-0.0547916	0.4233326	0.0010983	0.980495	-0.0194366	0.7708664
READ	0.2042762	0.0331698	0.1635918	0.1074482	0.0374904	0.7200245	0.0870552	0.4604069	0.0767425	0.5011343
SARC	-0.1124867	0.1754277	-0.1290234	0.1074482	-0.0797719	0.3995943	-0.1185223	0.1324912	-0.1069092	0.2062081
SKCM	0.0225762	0.7034461	0.0143197	0.7759886	0.0285554	0.6705504	0.0310377	0.6270036	0.0398959	0.5346906
STAD	0.1037024	0.0991276	0.0900614	0.1572234	0.0271823	0.7040324	-0.0067254	0.9607089	0.009932	0.9196035
TGCT	-0.202507	0.0470764	-0.3468759	9.13E-05	-0.1668536	0.1269975	-0.2151936	0.0363519	-0.121433	0.292204
THCA	0.2995669	2.07E-10	0.2495357	1.54E-07	0.3164221	1.07E-11	0.3257641	9.54E-13	0.3641347	8.39E-16
THYM	-0.0440169	0.7034461	0.0464199	0.7007478	0.0915202	0.55596	-0.0437391	0.7260001	0.0119869	0.9196035
UCEC	0.0021207	0.9819047	0.0343066	0.5654951	-0.0698097	0.2436035	-0.0992766	0.0681567	-0.0713541	0.2135498
UCS	-0.1036427	0.598794	-0.1389033	0.4658304	-0.0865958	0.6705504	-0.1245139	0.5335719	-0.1690433	0.397596
UVM	0.3212377	0.0209561	0.0810127	0.5937476	0.2966714	0.0376786	0.2226207	0.1257704	0.3469526	0.0092359

Cancer	HLA-E cor	HLA-E adj.p	HLA-F cor	HLA-F adj.p	HLA-G cor	HLA-G adj.p	TAP1 cor	TAP1 adj.p	TAP2 cor	TAP2 adj.p
ACC	-0.0561344	0.845666	-0.1305258	0.4192725	0.0995375	0.8058622	-0.0401168	0.8536119	0.1493427	0.480867
BLCA	0.1223969	0.0445342	0.13505	0.022889	0.1028436	0.1892507	0.1668963	0.0031685	0.175877	0.0047688
BRCA	0.1808996	2.02E-08	0.1460194	1.15E-05	0.0800343	0.0633185	0.1130595	0.0008579	0.1015385	0.0059582
CESC	0.0962576	0.2183416	0.0821335	0.3035488	0.0628986	0.6060061	-0.0094587	0.9148548	0.0652948	0.5364505
CHOL	0.3886744	0.0589077	0.2501931	0.2970556	0.2393822	0.456232	0.2157014	0.3589921	0.2223938	0.480867
COAD	-0.1156483	0.0445342	-0.17629	0.0008532	-0.0081938	0.9309991	-0.2090125	6.46E-05	-0.0849647	0.2518986
DLBC	0.016066	0.9136932	0.0566652	0.8259476	-0.0007599	0.9959102	-0.0649153	0.8323207	-0.0719713	0.8088806
ESCA	0.1061001	0.317064	0.0471836	0.7222377	-0.0521381	0.9055657	0.0214056	0.8827693	0.0107464	0.9311251
GBM	-0.0218662	0.9102452	-0.0421977	0.8060304	0.0288355	0.9236061	-0.2651683	0.0036979	-0.0955095	0.5364505
HNSC	-0.0119755	0.9102452	0.0591648	0.3220233	0.0552433	0.5221465	-0.0051859	0.9291371	-0.0188441	0.8344096
KICH	0.1022242	0.637034	-0.0256343	0.9312358	0.0169504	0.9345205	0.1114509	0.5660811	0.1243724	0.5599844
KIRC	-0.2221261	1.77E-06	-0.1282662	0.0120458	0.0278797	0.9055657	-0.0984747	0.0574668	-0.1170209	0.0382413
KIRP	-0.2487586	0.0001216	0.0910639	0.2799772	0.0986598	0.3240411	-0.082598	0.2920761	-0.0471361	0.6782374
LGG	-0.2653025	1.85E-08	-0.199528	3.95E-05	-0.1906775	0.0001727	-0.2487399	2.05E-07	-0.14323	0.0073621
LIHC	0.01753	0.9102452	-0.0922752	0.2023344	-0.0203621	0.9236061	-0.0730348	0.2920761	-0.0369893	0.7074313
LUAD	0.0091169	0.9126401	0.0289209	0.7222377	0.103338	0.108523	-0.0022531	0.9593201	0.0507705	0.5364505
LUSC	0.3387628	2.57E-13	0.3544331	1.13E-14	0.262896	4.60E-08	0.1944301	7.98E-05	0.2238839	1.65E-05
MESO	-0.266403	0.0445342	-0.2549027	0.0528823	-0.1915871	0.3018354	-0.1644674	0.2673297	-0.0778414	0.7074313
OV	0.0381498	0.7260711	-0.0520371	0.5625324	0.0104269	0.9309991	-0.1524503	0.0224406	-0.0282566	0.8088806
PAAD	-0.0126044	0.9126401	-0.016561	0.9312358	-0.020526	0.9236061	0.0455192	0.7519226	0.0749942	0.5599844
PCPG	-0.2865218	0.0005266	-0.2442859	0.0045967	-0.0246636	0.9236061	-0.0731973	0.5239215	0.0252727	0.8636514
PRAD	-0.0770914	0.2174228	-0.0178706	0.8259476	0.0050125	0.9345205	0.1376672	0.0075495	-0.0024134	0.9623684
READ	0.037194	0.845666	-0.0686176	0.5625324	0.0310079	0.9236061	-0.0771937	0.5239215	-0.0187905	0.8757897
SARC	-0.1126785	0.1991175	-0.2415712	0.0005549	-0.2344862	0.0013558	-0.267837	7.98E-05	-0.1650728	0.0382413
SKCM	0.0435906	0.6052343	0.0706033	0.2799772	-0.0299427	0.9055657	-0.0091615	0.9148548	-0.0455331	0.5599844
STAD	0.0433612	0.6052343	-0.067374	0.3220233	0.0096501	0.9309991	-0.2582576	1.27E-06	-0.1668658	0.0059582
TGCT	0.0576897	0.7157964	-0.2128468	0.0297374	0.1003191	0.5221465	-0.181745	0.0612315	-0.0790951	0.5599844
THCA	0.11052	0.0445342	0.3028676	5.87E-11	0.4511521	2.75E-25	0.3202725	5.29E-12	0.1812839	0.0007776
THYM	-0.086249	0.6052343	0.0878672	0.5438831	-0.0273977	0.9236061	0.0176332	0.9148548	-0.089916	0.5599844
UCEC	-0.0389677	0.6052343	-0.0042506	0.9447532	-0.01466	0.9236061	-0.1185777	0.0171608	-0.0262299	0.7690138
UCS	-0.2162302	0.2360002	-0.0587892	0.8259476	-0.0715582	0.9236061	-0.304965	0.0561949	-0.2803345	0.1386968
UVM	0.297023	0.0373077	0.3505626	0.0063672	0.1867323	0.3240411	0.4139241	0.000773	0.1647679	0.4118353

Cancer	TAPBP cor	TAPBP adj.p
ACC	-0.1807936	0.2216549
BLCA	0.1344183	0.0261809
BRCA	0.0434391	0.2855949
CESC	0.1793422	0.0081651
CHOL	0.0684685	0.7858083
COAD	-0.0813086	0.1826109
DLBC	-0.056231	0.7858083
ESCA	0.0862628	0.4226048
GBM	-0.0286612	0.7858083
HNSC	0.1920649	9.92E-05
KICH	0.1300087	0.4966555
KIRC	-0.0790939	0.1601456
KIRP	0.3690741	1.73E-09
LGG	-0.1649812	0.0013357
LIHC	-0.0133176	0.8186796
LUAD	0.0340199	0.6301044
LUSC	0.3792273	5.58E-17
MESO	-0.2619924	0.0406645
OV	-0.0445277	0.6301044
PAAD	0.0593622	0.6301044
PCPG	0.1643697	0.0675708
PRAD	0.1121466	0.0377553
READ	-0.0259551	0.7858083
SARC	-0.2060439	0.0050817
SKCM	0.0269014	0.7414436
STAD	0.0003702	0.9940008
TGCT	0.1963234	0.0427941
THCA	0.265364	1.59E-08
THYM	-0.2310299	0.037082
UCEC	0.0202652	0.7770752
UCS	-0.3450869	0.0311475
UVM	-0.0636896	0.7414436
