# Supplementary material for: Pan-Cancer Analysis Reveals the Multidimensional Expression and Prognostic and Immunologic Roles of VSTM2L in Cancer
Source: Front Mol Biosci. 2022 Jan 27;8:792154. doi: 10.3389/fmolb.2021.792154 (PMC8829123; doi:10.3389/fmolb.2021.792154)
Supplement: Supplementary file 7 [file DataSheet1.PDF]

Cancer	ULBP1 cor	ULBP1 adj.p	TNFSF9 cor	TNFSF9 adj.p	TNFSF4 cor	TNFSF4 adj.p	TNFSF18 cor	TNFSF18 adj.p	TNFSF15 cor	TNFSF15 adj.p
ACC	0.15756765	0.33097539	0.31991237	0.0095348	0.13342259	0.37384256	0.00130237	0.99761357	0.02421108	0.92880233
BLCA	0.20011282	0.00037461	0.18439731	0.00065498	0.3958326	1.87E-15	0.09561345	0.14836351	0.00646038	0.92880233
BRCA	0.13564285	8.46E-05	0.26236888	1.20E-17	0.1286703	9.33E-05	0.07453567	0.06700917	0.16502987	4.92E-07
CESC	-0.0510792	0.57419433	-0.0116407	0.87368126	0.07089544	0.36036498	0.0030917	0.99761357	0.27417636	7.34E-06
CHOL	-0.1943372	0.46557246	0.11788932	0.59817117	0.16808237	0.46733826	0.25433432	0.29515742	-0.1043143	0.75155647
COAD	-0.0242169	0.75651144	-0.3387156	9.31E-13	0.18336527	0.00028779	0.21423328	4.97E-05	0.03822532	0.63758651
DLBC	0.25868432	0.16854064	0.27073383	0.11844651	0.14828485	0.46591386	-0.0576075	0.84525572	-0.0591684	0.83580976
ESCA	0.1100091	0.28641608	-0.0770782	0.39604014	0.28808233	0.00027985	0.09948125	0.32344543	0.06439483	0.63758651
GBM	0.08388793	0.49188499	0.36662847	2.10E-05	-0.0772888	0.47216562	0.26568201	0.00722168	-0.1168615	0.35357742
HNSC	0.15269878	0.00231805	-0.0313389	0.59367129	0.24355347	1.74E-07	-0.0001312	0.99761357	0.21045451	7.34E-06
KICH	0.10245384	0.57449774	0.12174349	0.42602058	0.06389796	0.69743191	0.19292468	0.29515742	0.01488378	0.92880233
KIRC	0.19452963	8.46E-05	-0.0081089	0.87368126	0.02998794	0.63181474	-0.1317597	0.0131611	0.00997881	0.92880233
KIRP	-0.138725	0.05568144	-0.1084473	0.11844651	0.03859909	0.6389632	-0.1786489	0.0131611	-0.2829639	7.34E-06
LGG	0.16501163	0.00095135	0.42222323	2.01E-22	-0.060991	0.31723636	0.00269553	0.99761357	-0.0524442	0.4933654
LIHC	0.142421	0.02108392	0.12431823	0.03317203	0.01644611	0.81319976	-0.0073698	0.99761357	0.05203873	0.60474052
LUAD	0.09102291	0.09732942	0.05978829	0.26000654	-0.0332511	0.60196294	-0.1707874	0.00098191	0.03453766	0.64318232
LUSC	-0.1218362	0.02108392	-0.0030621	0.94549184	0.20114513	4.56E-05	-0.1041038	0.06700917	0.28290679	4.51E-09
MESO	0.39908508	0.00085794	0.3339287	0.00449401	0.31513815	0.00786292	-0.1622392	0.29515742	-0.1343376	0.47729061
OV	0.07139697	0.41000929	0.24911352	6.54E-05	0.11308534	0.1036307	0.13740501	0.06700917	0.0548398	0.62076551
PAAD	-0.0295566	0.771666	0.12155964	0.16803498	0.04668782	0.6389632	-0.0837345	0.39275099	0.01025888	0.92880233
PCPG	0.21377685	0.01542789	-0.2041766	0.01228134	0.15426305	0.08971942	0.03559507	0.79284613	-0.0815509	0.55021826
PRAD	0.09746307	0.08472354	-0.1778055	0.00026456	-0.0101507	0.85861794	0.00523105	0.99761357	-0.132284	0.01240063
READ	-0.0796926	0.49188499	-0.2555883	0.00273506	0.2578287	0.00227847	0.18029172	0.06700917	0.0642837	0.63758651
SARC	-0.0414807	0.67394938	-0.0171892	0.84612111	-0.1122805	0.14137985	-0.0356225	0.73219438	-0.0461858	0.65481633
SKCM	0.02086097	0.771666	0.13413208	0.00885068	0.05746777	0.36036498	0.05725849	0.35806421	0.07469306	0.28120219
STAD	0.04048706	0.57449774	-0.1384239	0.01050633	0.20973258	9.33E-05	0.26519512	8.29E-07	0.11570949	0.05249668
TGCT	0.0668243	0.57449774	-0.105226	0.28572505	0.52140319	1.05E-10	0.0968721	0.36659077	0.22495744	0.02053021
THCA	0.09283831	0.09671651	0.51596047	2.28E-34	0.40019911	2.13E-19	0.42292025	6.69E-22	0.25015106	2.12E-07
THYM	-0.0406457	0.771666	-0.1544274	0.1536107	-0.0189596	0.85861794	-0.1287558	0.30951656	-0.04476	0.78421169
UCEC	-0.0101396	0.8561037	-0.0675705	0.17709453	-0.005068	0.90603416	-0.0999478	0.06700917	-0.007665	0.92880233
UCS	0.05444646	0.771666	-0.0410941	0.84611379	0.33601244	0.02651588	0.25494064	0.14836351	0.12284853	0.6306354
UVM	0.54596924	6.49E-06	0.45138303	0.00012131	0.22444913	0.10073928	0.10694238	0.47597003	0.26690106	0.0513838

Cancer	TNFSF14 cor	TNFSF14 adj.p	TNFSF13B cor	TNFSF13B adj.p	TNFSF13 cor	TNFSF13 adj.p	TNFRSF9 cor	TNFRSF9 adj.p	TNFRSF8 cor	TNFRSF8 adj.p
ACC	-0.1921577	0.25651292	0.23982473	0.0831758	-0.1984907	0.18705758	-0.2356669	0.0859879	-0.1675755	0.24330453
BLCA	0.15960196	0.00608919	0.33828053	8.88E-11	0.09753786	0.12243435	0.27728397	1.21E-07	0.36797294	2.10E-13
BRCA	0.16244408	1.21E-06	0.10940097	0.00138877	-0.015078	0.72635248	0.17757603	4.04E-08	0.2379727	2.51E-14
CESC	0.00202584	0.97184635	0.03973816	0.65144547	0.16416416	0.01448351	0.00202413	0.97187001	0.12710202	0.05821207
CHOL	-0.1287001	0.69709114	0.31531532	0.14361636	0.14285714	0.56664883	0.44593204	0.0197339	0.37477477	0.0572172
COAD	0.08859687	0.17890831	0.03852117	0.58689384	-0.1583828	0.0033475	0.02944884	0.65181298	0.13807196	0.0094355
DLBC	0.03573369	0.91034402	-0.0320234	0.8961423	-0.121472	0.56664883	0.02040816	0.91332721	0.33933999	0.04574522
ESCA	0.14780362	0.14889536	0.11268906	0.2303765	0.11378644	0.25900712	0.17356431	0.0453571	0.25822372	0.00154799
GBM	0.07413439	0.63033027	-0.0509697	0.68582447	-0.0339217	0.75060365	-0.0697787	0.5398611	0.04420476	0.65270004
HNSC	0.04061058	0.63033027	0.10178238	0.05339376	0.21023281	1.26E-05	0.18960059	5.18E-05	0.22027238	2.97E-06
KICH	0.27401002	0.10396748	0.28688328	0.05339376	-0.0458412	0.75236203	0.19140529	0.22485653	0.22828753	0.13048056
KIRC	0.17734277	0.00030661	0.1897245	6.89E-05	-0.272319	2.16E-09	0.08587647	0.10560406	0.14432367	0.00277598
KIRP	-0.0864149	0.35527061	-0.1494158	0.03942128	0.5369605	9.28E-22	-0.3146671	2.91E-07	-0.0352548	0.65165157
LGG	-0.1922697	0.00010924	-0.1846262	0.00013956	0.03851457	0.56664883	-0.2459302	1.21E-07	-0.1790358	0.00024635
LIHC	0.0311753	0.73258807	0.00890986	0.90967588	0.13359724	0.03330349	-0.0289427	0.66103878	-0.0203103	0.73539274
LUAD	-0.0212865	0.78730364	-0.0098155	0.8961423	0.0335957	0.59572605	0.00762489	0.90836999	-0.0163472	0.73539274
LUSC	0.36710439	7.93E-16	0.28963676	7.74E-10	0.42641678	5.96E-22	0.32313019	4.89E-12	0.35154634	2.04E-14
MESO	0.01851717	0.91034402	-0.4550922	6.89E-05	-0.2652548	0.04008672	0.27823139	0.0259166	-0.067872	0.65165157
OV	0.18300873	0.00611828	0.10628854	0.14364317	0.02266892	0.75060365	-0.0367174	0.65181298	0.09914492	0.1617202
PAAD	-0.0629748	0.67054317	-0.103557	0.27956587	0.05034629	0.62913574	-0.1224073	0.20520346	-0.0735756	0.46810844
PCPG	-0.0477591	0.72163264	0.11799526	0.21647652	0.05346366	0.61255169	-0.0421251	0.66103878	-0.0271244	0.73539274
PRAD	-0.0089226	0.91034402	0.00616039	0.91377189	-0.0640982	0.30642022	0.02893429	0.65181298	-0.0448053	0.46810844
READ	0.10462223	0.37846427	0.14147608	0.14534206	-0.031362	0.75060365	0.07979883	0.49085101	0.08923408	0.40466548
SARC	-0.0596274	0.63033027	-0.1032718	0.19317393	-0.0179006	0.78550851	-0.1136193	0.14184456	0.09704606	0.21551171
SKCM	0.01517584	0.87357086	-1.91E-05	0.99966976	0.11029738	0.04752789	-0.013444	0.83356366	-0.0330654	0.61168813
STAD	0.17790191	0.00154406	0.04174104	0.58689384	-0.1528542	0.00796025	0.07825971	0.21220198	0.20449154	0.00017977
TGCT	-0.1315477	0.28953542	-0.3986506	4.36E-06	0.29440064	0.00145889	-0.3718121	1.60E-05	-0.0459594	0.65270004
THCA	0.16717328	0.00100827	0.23748188	7.84E-07	0.0456692	0.51964013	0.30195027	6.87E-11	0.03565774	0.5628235
THYM	0.04073596	0.79838564	-0.0207723	0.8961423	-0.2758386	0.00917127	-0.1307174	0.26907446	0.07824154	0.54571687
UCEC	0.02929018	0.70715683	-0.0392246	0.56795119	-0.1641372	0.00079016	0.02645232	0.65181298	0.09379391	0.06013628
UCS	-0.1863018	0.36726316	0.04913145	0.89581613	-0.1837568	0.32614115	-0.1265429	0.51592525	0.08004926	0.65165157
UVM	0.16114987	0.36066277	0.26565213	0.05339376	-0.0700891	0.6505623	0.36420486	0.00326019	0.43668972	0.00025644

Cancer	TNFRSF4 cor	TNFRSF4 adj.p	TNFRSF25 cor	TNFRSF25 adj.p	TNFRSF18 cor	TNFRSF18 adj.p	TNFRSF17 cor	TNFRSF17 adj.p	TNFRSF14 cor	TNFRSF14 adj.p
ACC	0.13300876	0.40429828	-0.1138754	0.43815799	-0.2568436	0.06375667	-0.1192662	0.44422828	-0.2007303	0.17905243
BLCA	0.30409342	4.73E-09	-0.0422438	0.52632574	0.23104732	2.40E-05	0.21649599	0.00013655	-0.0677315	0.24585978
BRCA	0.22545837	7.65E-13	0.16065684	1.13E-06	-0.045294	0.31096077	0.14250874	4.16E-05	-0.0134019	0.71031315
CESC	0.11569686	0.08627386	-0.1773336	0.00953746	-0.0518903	0.53842778	0.12567434	0.05588001	0.16219511	0.01976928
CHOL	0.0952381	0.80081641	-0.0175032	0.91929595	0.01312741	0.93942872	0.44182102	0.02243388	0.26537967	0.19625271
COAD	0.2111674	2.95E-05	0.08443109	0.16716427	0.00748556	0.91901919	0.08078018	0.14031832	-0.0753836	0.19371127
DLBC	0.0026053	0.9859785	0.07273122	0.75544075	0.01584889	0.93831298	-0.0638298	0.78407292	0.20201911	0.24585978
ESCA	0.19517645	0.02586589	0.04497176	0.67911383	-0.0458929	0.62947746	0.27550374	0.00084172	0.1788286	0.0495692
GBM	-0.0236219	0.96521616	0.24045073	0.01101848	0.20315495	0.04284434	0.08435827	0.44422828	-0.1185785	0.22204277
HNSC	0.25679558	2.63E-08	-0.0061204	0.91929595	0.11847333	0.02962889	0.1816452	0.00029807	0.17396076	0.00075451
KICH	0.10664969	0.63047068	0.16806354	0.30847691	0.32677515	0.02962889	0.27609986	0.05518274	0.00192049	0.98778964
KIRC	-0.1428643	0.0037651	0.04860443	0.37976682	0.27874929	7.64E-10	0.24571444	3.62E-07	-0.3083436	6.67E-12
KIRP	-0.1517163	0.02762375	0.37869285	1.02E-09	0.05632107	0.53842778	-0.1558234	0.02243388	0.23030812	0.00075451
LGG	-0.1155262	0.02652952	0.11491021	0.03267628	0.40723981	1.97E-20	-0.1015883	0.04940713	-0.0905978	0.11332667
LIHC	-0.0736759	0.27253335	0.05955589	0.37976682	-0.0558654	0.51483376	-0.0907274	0.14031832	0.01993641	0.73885174
LUAD	0.09244649	0.07977696	0.00745467	0.91929595	0.19232218	8.86E-05	-0.0935574	0.06434843	0.07023605	0.19371127
LUSC	0.37709454	8.97E-17	0.09963929	0.07917988	-0.0357206	0.5666537	0.18211099	0.00033002	0.42416667	1.07E-21
MESO	0.35297441	0.00354865	0.25544944	0.05647164	0.09590289	0.53842778	-0.0595839	0.72943376	-0.1415032	0.26357607
OV	-0.0136152	0.96521616	0.11021969	0.1403239	0.01601707	0.84460915	-0.0273452	0.77020184	0.09793965	0.19371127
PAAD	0.01547716	0.96521616	0.14779152	0.13813008	0.09192141	0.42100402	-0.105492	0.25583243	0.22921558	0.01157656
PCPG	-0.1549592	0.07977696	0.30638698	0.0002185	-0.0537158	0.59080421	0.05808678	0.56428932	0.08698318	0.31520821
PRAD	-0.0296582	0.75559686	-0.1383479	0.00953746	0.05848282	0.38519499	0.00957976	0.89604639	0.04466828	0.37160085
READ	0.161291	0.07977696	0.09059952	0.37976682	0.06530422	0.55613702	0.13483947	0.14031832	0.09892635	0.27303601
SARC	-0.0293477	0.85015024	0.09925854	0.220652	0.03155168	0.70006243	-0.0013423	0.98281489	-0.1450253	0.05940187
SKCM	-0.0060549	0.96521616	0.05499224	0.37976682	0.04389535	0.53842778	-0.0429307	0.48627359	0.12596707	0.02476683
STAD	0.11860082	0.0416926	-0.0133022	0.87446768	0.01657449	0.81819852	0.12672996	0.02439237	-0.031968	0.5733937
TGCT	0.01032757	0.96521616	-0.4365723	4.67E-07	-0.1943784	0.05276515	-0.213919	0.02286698	-0.0880128	0.35521474
THCA	0.00761003	0.96521616	0.16208597	0.00160348	0.31950715	3.05E-12	0.17806192	0.00035678	0.07150308	0.19371127
THYM	0.02578651	0.96521616	0.15081603	0.220652	0.25478853	0.02489732	0.24499313	0.02243388	0.14678797	0.19371127
UCEC	0.08154628	0.1087691	0.06243248	0.26456406	0.02663159	0.62947746	0.03455211	0.56108095	-0.069243	0.19371127
UCS	-0.0890589	0.75559686	-0.2094244	0.22464535	-0.1979518	0.31096077	0.05173535	0.80264681	-0.2510371	0.15898988
UVM	0.18258322	0.19092959	0.18077825	0.220652	0.42089594	0.00057857	-0.0088749	0.96176771	0.36066573	0.0067632

Cancer	TNFRSF13C cor	TNFRSF13C adj.p	TNFRSF13B cor	TNFRSF13B adj.p	TNFRSF9 cor	TNFRSF9 adj.p	TNFRSF8 cor	TNFRSF8 adj.p	TNFRSF4 cor	TNFRSF4 adj.p
ACC	0.07863726	0.70129252	-0.1061448	0.48529244	-0.2356669	0.0859879	-0.1675755	0.24330453	0.13300876	0.40429828
BLCA	0.18274892	0.00206179	0.25641269	1.21E-06	0.27728397	1.21E-07	0.36797294	2.10E-13	0.30409342	4.73E-09
BRCA	0.09743396	0.00809402	0.17548999	6.20E-08	0.17757603	4.04E-08	0.2379727	2.51E-14	0.22545837	7.65E-13
CESC	0.00685641	0.90491982	0.06287235	0.40637798	0.00202413	0.97187001	0.12710202	0.05821207	0.11569686	0.08627386
CHOL	-0.0520619	0.84195012	0.28632381	0.18092416	0.44593204	0.0197339	0.37477477	0.0572172	0.0952381	0.80081641
COAD	-0.0486255	0.52014373	0.1515357	0.00571072	0.02944884	0.65181298	0.13807196	0.0094355	0.2111674	2.95E-05
DLBC	0.04005645	0.84195012	-0.2002822	0.31324747	0.02040816	0.91332721	0.33933999	0.04574522	0.0026053	0.9859785
ESCA	0.22474129	0.00933891	0.17914508	0.045205	0.17356431	0.0453571	0.25822372	0.00154799	0.19517645	0.02586589
GBM	0.24773916	0.00933891	0.02363325	0.90539307	-0.0697787	0.5398611	0.04420476	0.65270004	-0.0236219	0.96521616
HNSC	0.16841309	0.00147385	0.12592189	0.01758728	0.18960059	5.18E-05	0.22027238	2.97E-06	0.25679558	2.63E-08
KICH	-0.0580321	0.79386935	0.21257929	0.18092416	0.19140529	0.22485653	0.22828753	0.13048056	0.10664969	0.63047068
KIRC	0.17392075	0.0010844	0.1969576	2.64E-05	0.08587647	0.10560406	0.14432367	0.00277598	-0.1428643	0.0037651
KIRP	0.01015438	0.88543138	-0.1580685	0.02797345	-0.3146671	2.91E-07	-0.0352548	0.65165157	-0.1517163	0.02762375
LGG	-0.0787745	0.19459017	-0.0695467	0.21827434	-0.2459302	1.21E-07	-0.1790358	0.00024635	-0.1155262	0.02652952
LIHC	-0.0623834	0.41935162	-0.062394	0.384274	-0.0289427	0.66103878	-0.0203103	0.73539274	-0.0736759	0.27253335
LUAD	-0.059041	0.3809966	-0.0158243	0.87290789	0.00762489	0.90836999	-0.0163472	0.73539274	0.09244649	0.07977696
LUSC	0.05603674	0.40100011	0.33439377	5.96E-13	0.32313019	4.89E-12	0.35154634	2.04E-14	0.37709454	8.97E-17
MESO	0.07651229	0.70129252	-0.061358	0.7385428	0.27823139	0.0259166	-0.067872	0.65165157	0.35297441	0.00354865
OV	-0.0275297	0.79386935	0.01470676	0.90539307	-0.0367174	0.65181298	0.09914492	0.1617202	-0.0136152	0.96521616
PAAD	-0.0280189	0.83489247	-0.0830665	0.40637798	-0.1224073	0.20520346	-0.0735756	0.46810844	0.01547716	0.96521616
PCPG	0.01897508	0.84195012	-0.0296808	0.86454582	-0.0421251	0.66103878	-0.0271244	0.73539274	-0.1549592	0.07977696
PRAD	-0.1032652	0.07058668	-0.009214	0.90539307	0.02893429	0.65181298	-0.0448053	0.46810844	-0.0296582	0.75559686
READ	-0.0440828	0.79005251	0.18551452	0.04775474	0.07979883	0.49085101	0.08923408	0.40466548	0.161291	0.07977696
SARC	0.08417965	0.3809966	-0.0764597	0.38116742	-0.1136193	0.14184456	0.09704606	0.21551171	-0.0293477	0.85015024
SKCM	0.04092512	0.57771551	0.00577174	0.94797822	-0.013444	0.83356366	-0.0330654	0.61168813	-0.0060549	0.96521616
STAD	0.26004127	3.08E-06	0.32741449	1.59E-10	0.07825971	0.21220198	0.20449154	0.00017977	0.11860082	0.0416926
TGCT	0.15351616	0.17346313	-0.1795207	0.06984295	-0.3718121	1.60E-05	-0.0459594	0.65270004	0.01032757	0.96521616
THCA	0.05644514	0.40100011	0.23783268	5.61E-07	0.30195027	6.87E-11	0.03565774	0.5628235	0.00761003	0.96521616
THYM	0.0482603	0.79386935	0.20398653	0.06782801	-0.1307174	0.26907446	0.07824154	0.54571687	0.02578651	0.96521616
UCEC	0.14700823	0.00460606	0.04305322	0.45106089	0.02645232	0.65181298	0.09379391	0.06013628	0.08154628	0.1087691
UCS	0.12354161	0.57582902	0.03038953	0.90539307	-0.1265429	0.51592525	0.08004926	0.65165157	-0.0890589	0.75559686
UVM	-0.3142464	0.01646878	-0.0061909	0.9565352	0.36420486	0.00326019	0.43668972	0.00025644	0.18258322	0.19092959

Cancer	STING1 cor	STING1 adj.p	TMIGD2 cor	TMIGD2 adj.p	RAET1E cor	RAET1E adj.p	PVR cor	PVR adj.p	NT5E cor	NT5E adj.p
ACC	-0.0985881	0.4304019	-0.2398341	0.13305302	0.2622444	0.04601546	0.44298929	0.00057943	0.13186465	0.37376809
BLCA	0.27735792	6.09E-08	0.08624245	0.25192223	-0.2090605	9.21E-05	0.26638851	1.87E-06	0.16738593	0.00211445
BRCA	0.13707611	1.44E-05	0.164221	8.62E-07	0.03019546	0.40908348	0.00545242	0.90174351	0.15959958	5.88E-07
CESC	0.17284053	0.00419759	0.01800876	0.91357905	-0.1580447	0.01398086	0.20528253	0.00183378	0.47052986	5.80E-17
CHOL	0.33693694	0.06590831	0.01338481	0.98665131	0.18511844	0.38585797	0.12303732	0.82552097	0.10527671	0.67644173
COAD	-0.0417805	0.4304019	-0.0573493	0.45738032	-0.1000166	0.06812068	0.18216903	0.0008826	-0.0407193	0.5305134
DLBC	0.08684325	0.58658785	0.13167607	0.59421344	-0.0768997	0.68961108	0.02713851	0.90174351	0.07283977	0.73164866
ESCA	0.15835829	0.04819343	0.16556057	0.1080513	-0.105178	0.2570087	-0.0851276	0.4985561	0.03422539	0.73164866
GBM	0.07636404	0.42199263	0.23791766	0.02041698	0.13674005	0.18380875	0.02417139	0.86050608	-0.1508216	0.10913989
HNSC	0.28442376	2.05E-10	0.0737712	0.26352619	-0.2670485	5.66E-09	0.09033503	0.11170003	0.04723918	0.4019214
KICH	0.16950391	0.2170446	0.24769062	0.16343795	0.14422445	0.35704498	0.04043461	0.86050608	0.24020708	0.09914457
KIRC	0.12207833	0.00794568	0.17402095	0.00053677	-0.191199	4.62E-05	0.18052419	0.00055196	0.00964461	0.84533403
KIRP	-0.1056117	0.09359043	0.14065749	0.08267362	-0.3126016	4.33E-07	0.0733783	0.448025	0.10964983	0.10913989
LGG	-0.2965512	4.95E-11	0.0440563	0.55283415	-0.0030594	0.94472783	0.14352879	0.00479032	0.09342635	0.07524455
LIHC	0.17074776	0.00174532	-0.0478419	0.59421344	-0.0815751	0.2030442	0.01492657	0.86050608	-0.0737782	0.24980922
LUAD	0.24581653	6.88E-08	-0.0022665	0.98665131	0.14004296	0.00384583	0.02026904	0.86050608	0.21876701	2.67E-06
LUSC	0.47900419	1.69E-28	0.32181053	6.22E-12	0.17533471	0.00031877	0.17086938	0.00097104	0.27635099	2.49E-09
MESO	0.37034337	0.00082863	-0.2613739	0.08267362	-0.6308231	7.74E-10	0.29809725	0.01680824	0.3747175	0.00116388
OV	0.11299345	0.07058631	-0.0027495	0.98665131	0.07437849	0.30254835	0.1045475	0.18006028	0.37486239	1.52E-10
PAAD	0.32719645	2.08E-05	0.06514974	0.59421344	0.26073957	0.00130206	0.2659176	0.00183378	0.282918	0.00045222
PCPG	-0.2481209	0.00144314	0.03533748	0.88084839	-0.2484327	0.00213066	0.11336693	0.30265548	0.03307431	0.73164866
PRAD	-0.1695928	0.00033679	-0.0721444	0.28755008	-0.1972425	4.62E-05	0.00590252	0.91842721	-0.2376923	5.30E-07
READ	-0.0655298	0.43413299	-0.0008828	0.99099354	-0.0179564	0.86145774	0.03252159	0.86050608	0.08935076	0.37376809
SARC	-0.0290115	0.65792127	0.00461009	0.98665131	-0.1029396	0.18603821	0.03115431	0.86050608	-0.099066	0.18503642
SKCM	0.30015575	1.94E-10	0.01810247	0.89698494	-0.0304598	0.6177038	0.01845595	0.86050608	-0.136494	0.00727465
STAD	0.27233568	6.88E-08	0.03302849	0.74403501	0.11301274	0.0473261	0.04284563	0.69813296	0.15247898	0.00525382
TGCT	0.58738433	3.70E-14	0.01896084	0.94278798	0.53863671	4.65E-11	-0.2003966	0.04289168	0.62479755	1.72E-16
THCA	0.377733	2.09E-17	0.21035032	2.25E-05	0.30073422	8.46E-11	0.07980674	0.18006028	0.45731352	4.54E-26
THYM	-0.3181471	0.00082863	0.0952705	0.5466122	0.03987605	0.73223619	-0.2663657	0.01190923	-0.0729287	0.57146909
UCEC	0.10569411	0.02169636	0.05545414	0.43587821	0.16115034	0.00057448	-0.130228	0.00926999	-0.0833757	0.09914457
UCS	0.05477055	0.68573375	0.02995526	0.94278798	0.21062527	0.2030442	-0.0600856	0.86050608	0.03156598	0.84533403
UVM	0.73087201	1.40E-13	-0.1754567	0.29883255	-0.0177139	0.89853056	0.14301453	0.448025	0.4347398	0.00022353

Cancer	MICB cor	MICB adj.p	LTA cor	LTA adj.p	KLRK1 cor	KLRK1 adj.p	KLRC1 cor	KLRC1 adj.p	IL6R cor	IL6R adj.p
ACC	0.0634372	0.64290106	-0.0227284	0.84240353	-0.2600292	0.07509237	-0.1642555	0.33660616	0.0617332	0.84125018
BLCA	0.19483365	0.00059609	0.29666907	9.83E-09	0.23373366	6.17E-05	0.07359762	0.33660616	0.00696251	0.98773104
BRCA	-0.0332405	0.41588058	0.20089994	3.53E-10	0.13761904	6.17E-05	0.11984378	0.00103716	0.10314542	0.00306046
CESC	0.14360332	0.03402868	-0.0154785	0.84240353	0.06223122	0.41160549	0.04219776	0.76286075	0.01126318	0.98773104
CHOL	0.22702703	0.32666853	0.33050193	0.0932997	0.37889318	0.07558874	0.14948466	0.71113099	0.35701416	0.11839317
COAD	-0.0860788	0.15803663	0.06456747	0.27091342	-0.1204747	0.04383101	-0.1476214	0.00784894	0.22879517	7.47E-06
DLBC	-0.1170213	0.58225512	0.15165002	0.41863605	-0.1138732	0.60815589	-0.029209	0.91218394	-0.0022796	0.98773104
ESCA	0.04470452	0.64199053	0.21481748	0.00948736	0.1835355	0.04957751	0.13225614	0.20777463	-0.0029699	0.98773104
GBM	-0.1097966	0.32666853	0.10218905	0.32119506	0.15456422	0.15049544	0.03111907	0.82992475	0.10050192	0.39350858
HNSC	0.13007262	0.0107797	0.19543986	4.57E-05	0.0197425	0.75440153	0.02996388	0.76286075	0.07711396	0.20476814
KICH	0.13474726	0.41588058	0.1711947	0.27091342	0.213696	0.19008794	0.17854534	0.33660616	-0.0898245	0.72803336
KIRC	0.12847366	0.0107797	0.12496703	0.01028457	0.09128606	0.10806601	0.16313949	0.00155013	-0.2848025	4.20E-10
KIRP	-0.0674449	0.40360837	-0.2214349	0.00057681	0.01118214	0.87140095	-0.1371016	0.06502083	-0.170546	0.01590374
LGG	-0.3193907	4.24E-12	-0.1672439	0.00057681	-0.1348421	0.01428844	0.00905699	0.91218394	-0.1643872	0.00117585
LIHC	-0.0685267	0.32666853	-0.1097368	0.06921312	-0.0893902	0.19008794	-0.0521925	0.63211866	0.01521187	0.98773104
LUAD	-0.0179621	0.73973255	0.00903283	0.84240353	-0.0630482	0.25513339	-0.0371959	0.71113099	0.02444896	0.84125018
LUSC	0.2842694	1.82E-09	0.33819109	2.87E-13	0.20461207	6.17E-05	0.15703226	0.00334983	0.15718053	0.00236243
MESO	0.32581833	0.01036488	0.10895239	0.42016049	-0.0729387	0.6369513	-0.3177003	0.01205648	-0.1233324	0.44362948
OV	-0.1053059	0.15803663	-0.0161896	0.84240353	0.0351637	0.6570033	-0.0337102	0.82794546	-0.0912766	0.23754343
PAAD	0.00785889	0.94035298	-0.0799971	0.41014821	-0.0284247	0.78405267	0.01058942	0.92790516	-0.0228025	0.98773104
PCPG	-0.0581669	0.58225512	0.07039417	0.44692207	0.00548641	0.94156638	-0.0617527	0.71113099	-0.1461721	0.15258645
PRAD	-0.1292865	0.01283996	0.05531288	0.32277738	-0.1184632	0.04383101	-0.1499725	0.00524583	-0.1692385	0.00117585
READ	0.00144683	0.98523975	0.16554624	0.06921312	0.05154423	0.6369513	-0.0420812	0.82934205	0.12598724	0.23508475
SARC	-0.2316398	0.00109503	-0.1201292	0.09641208	-0.0762181	0.33943846	-0.1524508	0.050417	-0.1173223	0.16819368
SKCM	0.07362522	0.23271398	-0.0115767	0.84240353	0.06884213	0.24451369	0.00418022	0.92790516	0.00077608	0.98773104
STAD	-0.1684391	0.00325516	0.15981434	0.00362482	0.08740598	0.18825117	-0.0054249	0.92790516	0.29752807	8.36E-09
TGCT	-0.4479577	9.03E-08	-0.3548833	4.77E-05	-0.302369	0.00135558	-0.2201609	0.02714528	-0.5566772	5.58E-12
THCA	0.25840139	4.39E-08	0.27676206	2.81E-09	0.06996768	0.21883594	0.31696125	9.70E-12	0.1189769	0.02882217
THYM	-0.0615459	0.61124231	0.25026043	0.01459329	0.08938815	0.47371567	0.03151023	0.83727422	0.03537051	0.96734834
UCEC	-0.0956169	0.06827303	0.0309755	0.53773013	0.01052967	0.84868959	-0.0199138	0.82934205	-0.0022661	0.98773104
UCS	-0.3400311	0.02971027	0.12085846	0.46312648	-0.1976277	0.24451369	-0.4093045	0.00784894	0.17636764	0.36075863
UVM	0.04280356	0.74334523	0.28657258	0.02344056	0.227719	0.12058398	0.21927095	0.15592919	-0.0192218	0.98773104

Cancer	IL6 cor	IL6 adj.p	IL2RA cor	IL2RA adj.p	ICOSLG cor	ICOSLG adj.p	ICOS cor	ICOS adj.p	HHLA2 cor	HHLA2 adj.p
ACC	0.02566038	0.84347376	-0.2860775	0.02824853	-0.2288705	0.09701835	-0.2473497	0.06992235	-0.2892758	0.03533943
BLCA	0.35329404	7.77E-12	0.33435467	8.21E-11	0.26596289	4.92E-07	0.25749101	1.33E-06	0.12621422	0.0357234
BRCA	0.13707839	4.04E-05	0.1921621	1.75E-09	0.13724238	3.93E-05	0.18483363	8.74E-09	0.11475686	0.0010924
CESC	0.16316655	0.01531936	0.07409351	0.33250112	-0.1864805	0.00380809	0.05566463	0.47399561	0.0803532	0.35750822
CHOL	0.18752816	0.54684782	0.52590257	0.00358985	0.25971686	0.20174913	0.34449023	0.09327787	0.52880739	0.00522409
COAD	0.04840345	0.54781424	0.03236943	0.69935771	0.00645689	0.91322336	0.0337449	0.62836824	0.09577834	0.11564604
DLBC	0.13775884	0.60947081	-0.0184542	0.90567611	0.27702996	0.1078504	0.00759878	0.95911923	0.09901144	0.69400369
ESCA	-0.0246048	0.79951793	0.17946732	0.03627796	0.02947955	0.72671989	0.16231189	0.06992235	0.00371504	0.97356992
GBM	0.04182917	0.741065	-0.1193563	0.25762802	0.15994532	0.10163552	-0.1248494	0.23644118	0.13692974	0.21636986
HNSC	0.03542236	0.66908076	0.16960203	0.00043841	0.26249776	2.26E-08	0.13529429	0.00866043	0.00255415	0.97356992
KICH	0.2960597	0.05264917	0.09734051	0.64717279	-0.1269609	0.39961626	0.31257535	0.03858823	0.05055592	0.8585669
KIRC	0.24423255	1.49E-07	0.21264083	4.84E-06	-0.1209107	0.01596186	0.07336676	0.19079051	-0.2295299	1.12E-06
KIRP	-0.2282455	0.00043911	-0.1046015	0.15857637	-0.2394373	0.00021655	-0.1020592	0.1838558	0.19011273	0.00570484
LGG	-0.0913213	0.09526234	-0.2431011	1.79E-07	-0.0549126	0.30432259	-0.1505347	0.00343929	0.21808562	5.67E-06
LIHC	0.09983877	0.12867844	-0.0766138	0.25762802	0.10480104	0.09701835	-0.1257468	0.04841014	-0.03595	0.69400369
LUAD	-0.0223215	0.741065	-0.0198674	0.75477565	0.0850513	0.10747973	-0.0511031	0.42957557	0.24227721	5.16E-07
LUSC	0.13483894	0.00996546	0.27329055	4.95E-09	0.29372837	7.97E-10	0.30776422	7.52E-11	0.14312014	0.0058582
MESO	0.12085369	0.54684782	0.30524167	0.01347298	-0.1194685	0.36052054	0.11790114	0.44515302	0.00360416	0.97356992
OV	0.25225204	5.86E-05	0.08691492	0.25762802	0.08109046	0.23574152	-0.0172774	0.84663528	-0.0705789	0.40377073
PAAD	-0.0478145	0.70736435	0.01731007	0.88443391	-0.0704643	0.422545	-0.0740824	0.47399561	0.09171427	0.40377073
PCPG	-0.1728918	0.05697353	-0.156556	0.08311042	-0.2746666	0.00073058	-0.0336857	0.79100413	0.16646317	0.07726939
PRAD	-0.1051979	0.05697353	0.01857091	0.75477565	-0.0321869	0.55715629	-0.0161811	0.82135869	0.01148149	0.91231047
READ	-0.0809216	0.54781424	0.08829876	0.41270998	0.23953276	0.00627391	0.03181851	0.80475144	0.02302733	0.91231047
SARC	-0.0485241	0.66908076	-0.0642736	0.46439835	0.08833576	0.23574152	-0.1443392	0.05683242	0.01091152	0.95666748
SKCM	-0.0306774	0.70736435	-0.0277866	0.75477565	0.09475292	0.09701835	0.01271532	0.84663528	0.07773722	0.21636986
STAD	0.01916974	0.77444292	0.02419729	0.75477565	0.12473271	0.02954381	0.00682916	0.93651079	-0.0044174	0.97356992
TGCT	-0.4461763	1.49E-07	-0.3526148	4.81E-05	-0.0931864	0.35407345	-0.3284573	0.00027124	0.54465652	2.32E-11
THCA	0.23789941	5.57E-07	0.33541147	3.02E-13	0.18871322	0.00012146	0.2892258	5.79E-10	-0.0336946	0.68943626
THYM	-0.0444309	0.741065	-0.0414473	0.75477565	0.04495451	0.67661922	0.00593791	0.95911923	0.07025505	0.68943626
UCEC	0.09118754	0.08882314	-0.0178193	0.75477565	0.10873705	0.02954381	-0.0200748	0.79100413	0.11818706	0.02294721
UCS	0.03850143	0.81699585	-0.1724786	0.33250112	0.08834586	0.57048274	-0.1007973	0.62836824	0.12450707	0.61934256
UVM	0.39818834	0.00112995	0.06144649	0.75477565	0.17754337	0.19188313	0.26924133	0.04841014	0.15258832	0.37182911

Cancer	ENTPD1 cor	ENTPD1 adj.p	CXCR4 cor	CXCR4 adj.p	CXCL12 cor	CXCL12 adj.p	CD276 cor	CD276 adj.p	CD86 cor	CD86 adj.p
ACC	0.26669912	0.03501276	0.39072541	0.00123561	-0.1800633	0.15106886	0.12210321	0.40531697	-0.1270448	0.4408974
BLCA	0.32703636	3.00E-10	0.32858394	2.00E-10	0.34763059	9.89E-12	0.2859426	2.33E-08	0.34416893	1.16E-11
BRCA	0.10710026	0.00146838	0.1550173	1.36E-06	0.15594285	1.15E-06	0.18556732	4.43E-09	0.11535228	0.00083963
CESC	0.1649419	0.00952924	0.19792731	0.00152757	0.11307927	0.07698841	0.27642298	4.52E-06	-0.0012567	0.98253372
CHOL	0.22316602	0.30522785	0.31917632	0.11554051	0.27232947	0.15106886	-0.2646075	0.18286785	0.39124839	0.05776736
COAD	0.26474318	8.75E-08	-0.0299197	0.73449427	0.32267874	1.47E-11	0.07186581	0.18458165	0.04596644	0.50202905
DLBC	-0.0239904	0.91869816	-0.0664351	0.84347344	0.04548415	0.7783198	-0.0820669	0.7240243	0.19431177	0.32987857
ESCA	0.2874891	0.00032231	0.22814147	0.0051092	0.2039536	0.01339857	0.01474736	0.86366907	0.0511296	0.69922095
GBM	0.0142368	0.91869816	-0.1199221	0.24313611	0.13058381	0.15106886	-0.1640666	0.07766061	-0.0366826	0.78835724
HNSC	0.22682758	1.30E-06	0.27125892	2.58E-09	0.1918212	4.53E-05	0.13480218	0.00539921	0.16154054	0.00120181
KICH	0.07523302	0.66455733	0.12481082	0.53005712	0.30805039	0.02635319	0.22814141	0.10466763	0.19916709	0.24008055
KIRC	0.00054775	0.98993412	0.03808897	0.58485778	0.0286768	0.53562649	0.27087428	2.72E-09	0.11096472	0.04142203
KIRP	0.00488907	0.95787907	-0.0105671	0.95310788	-0.1191918	0.07089422	0.2218772	0.00048439	0.03705805	0.70617745
LGG	-0.183897	0.00013703	-0.2862528	4.61E-10	-0.0948954	0.0593191	-0.3587645	1.62E-15	-0.3141237	5.59E-12
LIHC	0.13221518	0.02272727	0.00067164	0.99923719	0.16677406	0.00361101	0.04673942	0.5094291	-0.0298226	0.72173555
LUAD	-0.0314124	0.65778656	0.02476005	0.76674621	0.04254151	0.38318998	0.11550337	0.02047165	0.01881612	0.78835724
LUSC	0.2954535	3.00E-10	0.35449732	1.12E-14	0.17578978	0.00025434	0.13256912	0.00737216	0.32473288	3.64E-12
MESO	0.2778122	0.02039982	0.16452213	0.23239702	0.09302326	0.43495484	0.48673179	7.78E-06	0.08822993	0.61692724
OV	0.05842737	0.47803873	-0.2046272	0.00122474	0.23552252	0.00012565	0.12136048	0.0661367	0.12822585	0.07318374
PAAD	-0.0458645	0.66455733	-0.0501601	0.73449427	-0.2027389	0.01527782	0.14796937	0.08012197	-0.0479652	0.70617745
PCPG	-0.2266387	0.00663043	-0.2826503	0.00046247	-0.1539615	0.06698049	-0.1482606	0.08012197	-0.0416874	0.72173555
PRAD	-0.1484693	0.00296491	-0.0991877	0.06718156	-0.2207968	3.24E-06	0.19353842	5.46E-05	-0.0489309	0.44124973
READ	0.11655069	0.23445012	7.48E-05	0.99923719	0.15200399	0.07781805	0.03191295	0.80101289	0.1060018	0.32987857
SARC	0.00997989	0.91869816	0.03888281	0.73449427	0.13438139	0.0593191	0.02218422	0.80101289	-0.1456633	0.05776736
SKCM	-0.1315902	0.00994597	0.0050658	0.9866893	0.00449323	0.92252329	-0.0152735	0.80101289	-0.0177475	0.80097184
STAD	0.30618779	2.48E-09	0.30229179	2.58E-09	0.35225685	5.76E-12	0.18542229	0.00048439	0.08202148	0.23795331
TGCT	0.05351527	0.66455733	0.07973154	0.53136677	0.53866038	1.47E-11	0.47788968	4.43E-09	-0.3104867	0.00083963
THCA	-0.0616296	0.27506142	0.09377805	0.08097341	-0.0508801	0.30529301	0.32790063	6.38E-13	0.26191954	1.98E-08
THYM	-0.0850128	0.50843714	-0.0237725	0.93719882	0.37903327	7.85E-05	-0.2061602	0.04775952	-0.12055	0.32987857
UCEC	-0.0232027	0.69276619	-0.0820053	0.11554051	0.22910482	4.24E-07	-0.014906	0.80101289	-0.0078864	0.89921733
UCS	-0.0418719	0.86528596	0.23139746	0.15865039	0.37691211	0.01027338	0.30464091	0.04466769	0.00570391	0.98253372
UVM	-0.5125645	7.80E-06	0.22512893	0.09926001	0.13398969	0.29505621	0.3754571	0.0017131	0.24428036	0.07728739

Cancer	CD80 cor	CD80 adj.p	CD70 cor	CD70 adj.p	CD48 cor	CD48 adj.p	CD40LG cor	CD40LG adj.p	CD40 cor	CD40 adj.p
ACC	-0.0415747	0.81908097	-0.0159264	0.97527863	-0.2968354	0.02871677	-0.2765395	0.04540721	0.1010224	0.50092114
BLCA	0.32481625	2.36E-10	0.28222519	6.57E-08	0.30350152	5.14E-09	0.19265221	0.00051405	0.06901871	0.27805622
BRCA	0.12871178	0.00012356	0.19662671	6.35E-10	0.18012003	1.78E-08	0.20181534	2.85E-10	0.17315029	7.47E-08
CESC	0.01853359	0.81908097	-0.0060531	0.97527863	0.03133863	0.65000246	0.07759006	0.28130147	0.0247111	0.703062
CHOL	0.16138996	0.68524157	0.35871034	0.07454994	0.44633205	0.02708433	0.42419562	0.03609489	0.3027027	0.14547211
COAD	-0.024902	0.81908097	-0.0076312	0.97527863	0.05073821	0.38421095	0.11288773	0.04814664	0.22286314	8.32E-06
DLBC	-0.0474381	0.81908097	0.08250109	0.76960914	-0.0004342	0.99766297	0.17206285	0.34605519	0.15816327	0.4192101
ESCA	0.0541583	0.72793848	0.11136045	0.20576556	0.19844964	0.02708433	0.22214746	0.00949084	0.15852697	0.06920802
GBM	-0.0405995	0.81908097	-0.0415618	0.7772066	-0.1525471	0.14064289	-0.1371376	0.19148351	0.04305215	0.66357691
HNSC	0.14932385	0.00275588	0.15112242	0.00303687	0.1033899	0.05181367	0.13578646	0.00833382	0.2664021	1.25E-08
KICH	0.16804442	0.44356061	0.40493427	0.00372379	0.23887109	0.14064289	0.18083597	0.26583194	0.20444843	0.18976916
KIRC	0.10180442	0.06242178	0.10117295	0.05193878	0.05968345	0.29366146	0.088781	0.09522712	-0.151655	0.00147499
KIRP	-0.2374742	0.00025169	-0.1342052	0.05564851	-0.035735	0.63110817	0.07324792	0.3286585	0.34817364	1.46E-08
LGG	-0.229554	1.35E-06	-0.0429668	0.48015013	-0.2972801	2.19E-10	-0.1591492	0.00141949	-0.2044514	1.42E-05
LIHC	-0.0476818	0.68524157	-0.0410741	0.59341471	-0.0447391	0.52026456	-0.0232252	0.77137604	-0.039467	0.5606255
LUAD	0.00196437	0.96452947	-0.004072	0.97527863	-0.0613953	0.29366146	-0.0288818	0.64140807	0.02573444	0.65894769
LUSC	0.30198283	1.01E-10	0.35931058	2.06E-15	0.27638778	5.14E-09	0.36873464	5.59E-16	0.38172856	3.18E-17
MESO	0.12123642	0.61956072	0.33066633	0.00781868	0.04082525	0.74456013	-0.028687	0.83679207	0.16517825	0.22960817
OV	0.02580806	0.81908097	0.09327437	0.18284803	0.03428119	0.63110817	0.01543057	0.83679207	-0.0789608	0.27805622
PAAD	-0.0232231	0.81908097	0.11312855	0.20576556	-0.0881907	0.35617975	-0.0914842	0.33072724	0.10211119	0.27805622
PCPG	-0.0237577	0.81908097	-0.0227236	0.92291606	-0.0519357	0.60932711	0.02766103	0.81332053	0.09711817	0.29752307
PRAD	-0.0170796	0.81908097	0.04319172	0.48015013	-0.0659064	0.28384903	-0.0335615	0.60652344	-0.2510883	1.07E-07
READ	0.05605671	0.72793848	-0.018578	0.95554472	0.06091519	0.56207482	0.10675613	0.28130147	0.33538575	3.64E-05
SARC	-0.208525	0.00286342	-0.1123676	0.12812799	-0.0873994	0.29366146	-0.0121005	0.86772682	0.02673071	0.703062
SKCM	-0.0217365	0.81908097	0.1163419	0.03541848	0.0500804	0.38421095	0.02598813	0.69539893	0.00580834	0.89995212
STAD	-0.0477907	0.68524157	-0.0242873	0.7772066	0.17842574	0.00147995	0.19851634	0.00031009	0.16902191	0.00167594
TGCT	-0.3942806	4.77E-06	-0.168349	0.07892743	-0.3949953	3.78E-06	-0.3344632	0.00022997	-0.260184	0.00347249
THCA	0.30464357	8.64E-11	0.49243318	7.61E-31	0.15926787	0.00154602	0.18763865	0.00020368	0.0839424	0.12299538
THYM	-0.0700951	0.72793848	0.00568095	0.97527863	0.13875964	0.27510425	0.14100979	0.24795157	-0.3075075	0.0018124
UCEC	0.0212704	0.81908097	0.12776122	0.01123159	0.02578861	0.63110817	0.07272327	0.19148351	0.13261155	0.00479811
UCS	0.14036395	0.66151935	-0.2605821	0.09573575	0.02313975	0.88650139	0.01956088	0.88516616	0.11083744	0.5313045
UVM	-0.0868208	0.72793848	0.47619268	5.35E-05	0.20417253	0.15394481	0.17064679	0.24795157	-0.1068917	0.48887711

Cancer	CD28 cor	CD28 adj.p	CD27 cor	CD27 adj.p	VSIR cor	VSIR adj.p	BTNL2 cor	BTNL2 adj.p
ACC	-0.2079484	0.12555716	-0.3319864	0.00932998	-0.0424051	0.71398112	-0.0371968	0.90283209
BLCA	0.30563471	5.72E-09	0.29785798	1.12E-08	0.1077984	0.058948	-0.0293835	0.76409399
BRCA	0.18071208	2.10E-08	0.19735972	1.61E-09	0.19581176	7.65E-10	0.08647487	0.02344338
CESC	0.08925878	0.19073063	0.0635886	0.38193775	0.10234631	0.10936971	0.04134185	0.724916
CHOL	0.2952381	0.13990241	0.38404118	0.04883754	0.2983269	0.1096793	0.2877184	0.20918086
COAD	0.13825652	0.00931379	0.09752991	0.07775368	0.11869517	0.02447664	0.0700127	0.29918454
DLBC	0.07425098	0.70397457	-0.1061659	0.54016462	0.16804168	0.30737861	-0.315183	0.10584142
ESCA	0.23113984	0.00516036	0.28686176	0.00050213	0.02712558	0.71398112	0.14326104	0.1591594
GBM	0.02929449	0.7775699	-0.0075356	0.9302863	0.22219653	0.01539129	0.20454391	0.05603245
HNSC	0.18275144	0.00013299	0.14300398	0.00467487	0.15588371	0.00116804	0.06106984	0.32709577
KICH	0.05945162	0.70596938	0.29819746	0.0429154	0.11477001	0.38791226	-0.0203916	0.91673816
KIRC	0.14484098	0.00289892	0.13884877	0.00483512	-0.03995	0.38791226	-0.0126176	0.90745488
KIRP	-0.106212	0.12893514	-0.0732912	0.31608413	0.18651097	0.00437019	0.19542506	0.00820118
LGG	-0.178198	0.00020833	-0.1409264	0.00483512	-0.0937557	0.06330244	0.22218058	1.37E-05
LIHC	-0.1183398	0.05656231	-0.0878331	0.16573434	0.23193223	2.83E-05	0.05769997	0.46543123
LUAD	0.0122739	0.80109398	-0.011856	0.82987717	0.07734028	0.1096793	-0.0301081	0.73391704
LUSC	0.34123236	1.59E-13	0.26999241	1.12E-08	0.39864122	6.27E-19	0.08173272	0.18016601
MESO	0.06304221	0.66100371	0.11556827	0.38193775	-0.5957571	9.20E-09	0.00287313	0.97892932
OV	0.16702011	0.01013674	-0.0201335	0.80755068	0.32262466	6.05E-08	0.01314409	0.91083728
PAAD	-0.0748101	0.42613698	-0.1044191	0.28238517	0.2330876	0.00482679	-0.1275288	0.20918086
PCPG	-0.1380021	0.12555716	0.1025722	0.28238517	-0.3160181	5.86E-05	-0.0614648	0.68191921
PRAD	-0.0325176	0.58860125	-0.0039301	0.9302863	-0.204129	2.19E-05	-0.1310852	0.02255456
READ	0.08982823	0.34449298	0.16284671	0.07775368	0.11215903	0.18779719	0.03246762	0.84743531
SARC	-0.1215749	0.11158061	-0.0667285	0.38193775	-0.1269858	0.07410108	0.13795822	0.10584142
SKCM	0.01462671	0.79109411	0.01490424	0.80755068	0.08269546	0.10936971	0.02763175	0.76409399
STAD	0.22185985	3.36E-05	0.20765639	0.00016057	0.3699586	1.32E-13	0.16617829	0.00820118
TGCT	-0.3511036	6.04E-05	-0.2958336	0.00135639	0.19911463	0.03068533	0.2518048	0.01504765
THCA	0.22420118	3.20E-06	-0.059896	0.28363371	-0.0829149	0.09853753	-0.0224938	0.79051041
THYM	0.11632058	0.30489343	0.21282728	0.04883754	0.09204806	0.37335973	-0.1341711	0.30318932
UCEC	0.01081608	0.80109398	0.0361576	0.48428951	0.26808676	2.00E-09	0.2080772	1.91E-05
UCS	0.17759917	0.28657999	-0.1263288	0.45041624	0.13080114	0.37958221	0.1668035	0.39076441
UVM	0.21570558	0.11505154	0.38846695	0.00184863	0.39556962	0.0010238	-0.2376074	0.11271618
