# Supplementary material for: Pan-Cancer Analysis Reveals the Multidimensional Expression and Prognostic and Immunologic Roles of VSTM2L in Cancer
Source: Front Mol Biosci. 2022 Jan 27;8:792154. doi: 10.3389/fmolb.2021.792154 (PMC8829123; doi:10.3389/fmolb.2021.792154)
Supplement: Supplementary file 8 [file DataSheet5.PDF]

CancerType	TMB r	TMB *P*	MSI r	MSI *P*
ACC	0.3157991	0.0045814	0.1250746	0.2720792
BLCA	0.0512719	0.3015368	0.0791195	0.1105482
BRCA	0.0583991	0.0684868	0.0426483	0.1711978
CESC	-0.119652	0.0431859	0.0034046	0.9530152
CHOL	-0.0712354	0.6797141	-0.214157	0.2089866
COAD	-0.3749493	1.15E-14	-0.2107465	1.13E-05
DLBC	0.1043148	0.5375686	0.0016284	0.9912356
ESCA	-0.0332734	0.67617	-0.1017118	0.200616
GBM	-0.1532573	0.0629353	0.0217069	0.7913539
HNSC	-0.1515021	0.0007478	-0.0045173	0.9200657
KICH	0.0430422	0.7335239	0.0539285	0.6696268
KIRC	-0.004681	0.9322848	-0.0435703	0.4266924
KIRP	0.1520682	0.011122	0.0537877	0.3656218
LAML	-0.0997453	0.4366912	-0.1495033	0.2236683
LGG	-0.1776199	6.40E-05	0.0656049	0.139781
LIHC	-0.1165125	0.0272846	-0.0176755	0.735055
LUAD	-0.0090892	0.8388634	0.0287087	0.5172996
LUSC	-0.1023485	0.0237534	-0.0332905	0.4608197
MESO	0.0887755	0.4365601	0.245023	0.0265104
OV	-0.0631503	0.2993907	0.039777	0.5135906
PAAD	0.0906676	0.2682224	-0.0158153	0.8354391
PCPG	0.0404169	0.5932581	0.1002162	0.183194
PRAD	0.2377283	1.28E-07	-0.0032422	0.9426408
READ	-0.2920953	0.0006777	-0.0699178	0.3920327
SARC	-0.1652977	0.0111508	-0.1218483	0.0528999
SKCM	-0.1797259	9.74E-05	0.0854443	0.0647649
STAD	-0.4351249	1.97E-18	-0.3099529	9.03E-10
TGCT	0.1029738	0.2177563	0.2408231	0.0029915
THCA	0.1186647	0.0091159	0.0952487	0.0348592
THYM	-0.2466223	0.0073507	-0.1206314	0.1931899
UCEC	-0.2178411	4.65E-07	-0.1043257	0.0154865
UCS	-0.280898	0.035988	-0.0591271	0.6651119
UVM	0.0367705	0.746075	0.2039092	0.0696428
